# Lifestyle, gene gain and loss, and transcriptional remodeling cause divergence in the transcriptomes of *Phytophthora infestans* and *Pythium ultimum* during potato tuber colonization

**DOI:** 10.1186/s12864-017-4151-2

**Published:** 2017-10-10

**Authors:** Audrey M. V. Ah-Fong, Jolly Shrivastava, Howard S. Judelson

**Affiliations:** Department of Plant Pathology and Microbiology, University of California, Riverside, CA 92521 USA

**Keywords:** Plant pathogen, RNA-seq, Evolution, Gene family, Oomycete, Regulatory subfunctionalization, Comparative genomics

## Abstract

**Background:**

How pathogen genomes evolve to support distinct lifestyles is not well-understood. The oomycete *Phytophthora infestans,* the potato blight agent, is a largely biotrophic pathogen that feeds from living host cells, which become necrotic only late in infection. The related oomycete *Pythium ultimum* grows saprophytically in soil and as a necrotroph in plants, causing massive tissue destruction. To learn what distinguishes their lifestyles, we compared their gene contents and expression patterns in media and a shared host, potato tuber.

**Results:**

Genes related to pathogenesis varied in temporal expression pattern, mRNA level, and family size between the species. A family’s aggregate expression during infection was not proportional to size due to transcriptional remodeling and pseudogenization. *Ph. infestans* had more stage-specific genes, while *Py. ultimum* tended towards more constitutive expression. *Ph. infestans* expressed more genes encoding secreted cell wall-degrading enzymes, but other categories such as secreted proteases and ABC transporters had higher transcript levels in *Py. ultimum*. Species-specific genes were identified including new *Pythium* genes, perforins, which may disrupt plant membranes. Genome-wide ortholog analyses identified substantial diversified expression, which correlated with sequence divergence. Pseudogenization was associated with gene family expansion, especially in gene clusters.

**Conclusion:**

This first large-scale analysis of transcriptional divergence within oomycetes revealed major shifts in genome composition and expression, including subfunctionalization within gene families. Biotrophy and necrotrophy seem determined by species-specific genes and the varied expression of shared pathogenicity factors, which may be useful targets for crop protection.

**Electronic supplementary material:**

The online version of this article (10.1186/s12864-017-4151-2) contains supplementary material, which is available to authorized users.

## Background

A key issue in plant-microbe interactions is understanding what distinguishes different pathogenic lifestyles. These range from biotrophic relationships in which the pathogen feeds from living host cells, to necrotrophic associations in which the microbe feeds on nutrients released from killed cells [1]. Some pathogens can switch between biotrophic and necrotrophic growth, or grow as saprophytes on organic debris. Events such as gene duplication, loss, regulatory subfunctionalization, neofunctionalization, and horizontal gene transfer are contributors to the diversification of such behaviors by microbes [2, 3].

Genomic, transcriptomic, and other analyses have identified many factors that underlie plant pathogen lifestyles. These include effectors that suppress host defenses during biotrophic growth, enzymes that defeat antimicrobial compounds produced by the host, cell wall-degrading enzymes (CWDEs), and transporters for acquiring nutrients. Attributes specific to each trophic type have remained elusive, however. While defense-suppressing effectors are commonly thought to be specific to biotrophs, some necrotrophs also suppress plant immunity [4]. There is also no general correlation between lifestyle and the number of CWDEs encoded by a pathogen [5–7]. Instead, biotrophs and necrotrophs may be distinguished by the timing or level of expression of shared factors [8–10].

One limitation of many biotroph-necrotroph comparisons is that they examine pathogens on different hosts, which may present the microbe with distinct physical or chemical signals [11, 12]. In the current study, we reduce such complications by studying *Phytophthora infestans* and *Pythium ultimum* on a common host, potato tubers*. Phytophthora* and *Pythium* are sister taxa in the peronosporalean lineage of oomycetes, and a responsible for blights, rots, and damping-off diseases of thousands of important plant species [13]. *Ph. infestans* is the notorious Irish Famine pathogen. Its disease, late blight, occurs on tubers when the pathogen enters natural openings or wounds. *Ph. infestans* is described as a hemibiotroph, since host necrosis occurs only near the end of the life cycle. *Py. ultimum* also infects tubers through wounds but is a necrotroph. Its disease, potato leak, is characterized by dark lesions that culminate in watery rotted tissue. Other distinctions are that only *Py. ultimum* can persist in nature as a saprophyte, only *Py. ultimum* has a broad host range, and only *Ph. infestans* forms abundant sporangia, which appear late in infection. Sporangia release zoospores, which initiate most infections in late blight. While *Py. ultimum* sometimes makes terminal hyphal swellings, it is unclear if they release zoospores [14, 15].

Oomycete genomes contain many fast-evolving regions and gene families [16]. Studies in other kingdoms have shown that family expansions are often associated with changes in protein function, pseudogenization, or regulatory subfunctionalization i.e. altered transcription [2]. While there is moderate synteny between the genomes of *Ph. infestans* and *Py. ultimum*, the former is larger (237 vs. 43 Mb) with more predicted genes (17,797 vs. 15,290; [17, 18]). Examples of expanded families in *Ph. infestans* and *Py. ultimum* are RXLR effectors and secreted proteases, respectively. Little is known of how changes in gene families are manifested in their temporal patterns or levels of expression in oomycetes [19]. Genome-wide expression studies of *Ph. infestans* and *Py. ultimum* during plant infection are also limited. These include a study of *Py. ultimum* growing on *Arabidopsis* seeds in dilute V8 juice [17] and *Ph. infestans* on tomato and potato leaves [18, 20], although the latter lacked sufficient sequence depth at most timepoints to measure most genes.

Here, we use deep sequencing to compare the transcriptomes of *Ph. infestans* and *Py. ultimum* on potato tubers and artificial media. Infection-induced genes were identified in both species, but global analyses and those focused on specific functional groups revealed divergence in stage-specific expression patterns and mRNA levels. Differences between the hemibiotroph and necrotroph could be attributed to at least five phenomena: transcriptional remodeling that affects the level of expression; changes in the timing of expression; expansion and contraction of gene families; species-specific genes; and selective pressure on gene families to support biotrophy or necrotrophy.

## Results

### Comparisons of disease development

**Fig. 1:**
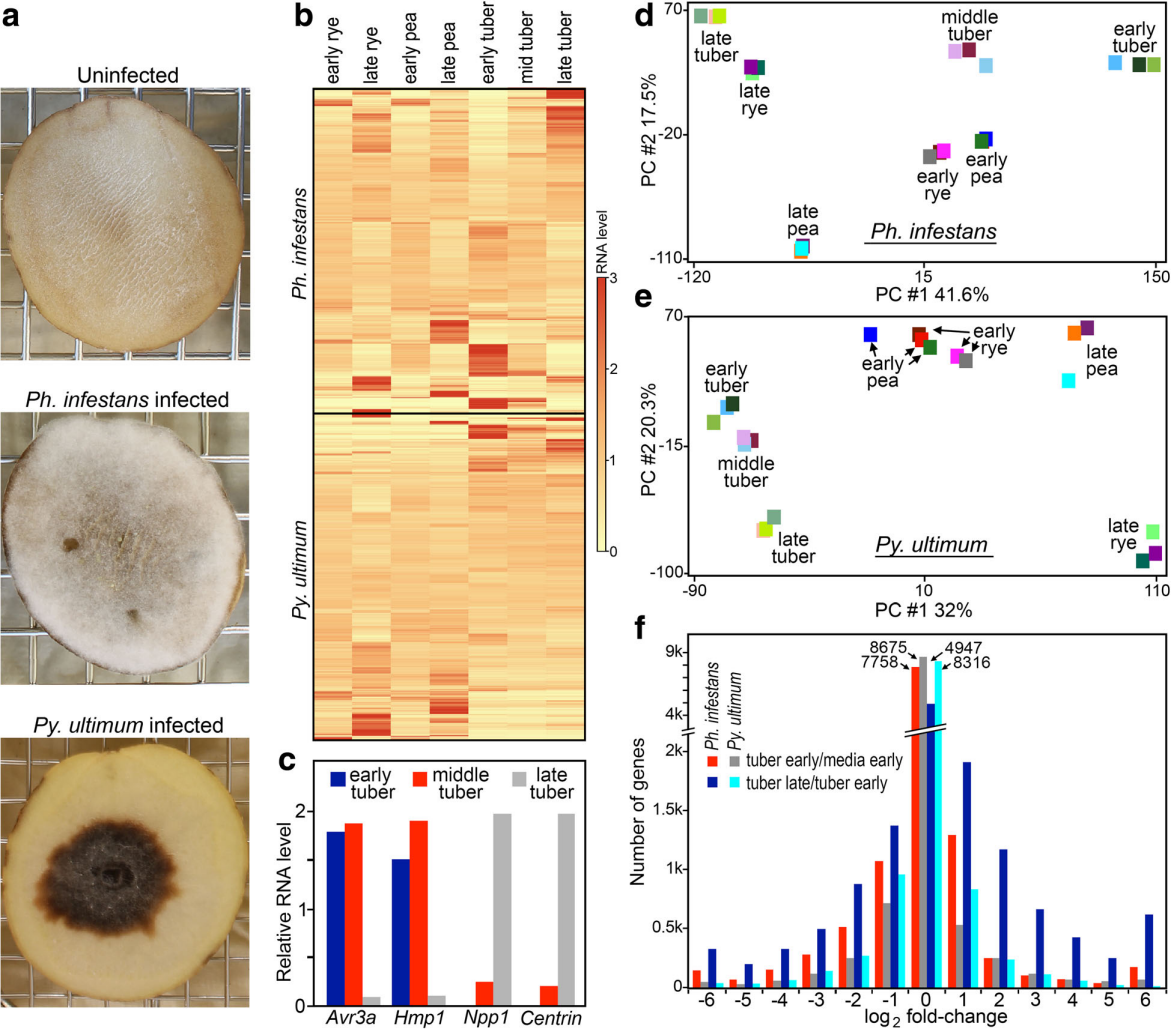
Overview of expression data. **a** images of uninfected tuber, tuber infected with *Ph. infestans* at 4 dpi, and tuber infected with *Py. ultimum* at 1.5 dpi. *Ph. infestans*-infected tubers are asymptomatic at 1.5 dpi. **b** Heatmaps of hierarchical clustered TMM-normalized data from artificial media and plant samples for *Ph. infestans* (top) and *Py. ultimum* (bottom). Only genes with CPM ≥1 in at least one condition are shown. **c** Expression of stage-specific markers in early, middle and late tuber samples of *Ph. infestans.* **d** and **e** Principal component analysis (PCA) displaying intrinsic biological variation between replicates of *Ph. infestans* and *Py. ultimum* samples. **f** log_2_ fold-change ratios in comparisons of early tuber versus early media, and late versus early tuber

Conditions for examining gene expression were established by testing strategies for inoculating tuber slices with zoospores of *Ph. infestans* and hyphae of *Py. ultimum*. These are the main propagules that infect tubers in soil or storage [15, 21]. With *Ph. infestans,* fairly synchronous infections were obtained by spreading zoospores over each tuber slice. Consistent with the biotrophic nature of the early to middle stages of the disease cycle, the first macroscopic evidence of infection were a few surface hyphae on the non-inoculated side of the slice at 2.5 dpi (days post-infection). Moreover, microscopic analyses performed between 1.5 and 2.5 dpi revealed haustoria. By 3.5 dpi, hyphae and a few sporangia were observed on both sides of the tuber. By 4 dpi, more surface hyphae and sporangia were evident as was slight darkening of host tissue (Fig. 1a). An alternative strategy for infecting tubers by applying a single drop of zoospores proved unsatisfactory. This resulted in lesions that expanded nonuniformly, and often sporulated first at patches distant from the inoculation site.

Disease progression was more rapid with *Py. ultimum.* When tubers were inoculated with a plug of hyphae, an expanding zone of darkened host tissue appeared by 0.5 dpi. At 1.5 dpi, host tissues were reddish brown on the outer periphery of the lesion, brown in the middle-aged part, and black in the oldest part (Fig. 1a). Sparse hyphae were seen on the surface of the brown and black zones. Hyphae were found within the tuber in the discolored region, but few in the non-discolored region. An alternative strategy for infecting tubers by applying hyphae to the entire slice proved unsatisfactory, since movement of the pathogen through the tuber was not uniform.

Based on the above, we collected material for RNA-seq by spreading *Ph. infestans* zoospores on tuber slices and harvesting 1.5, 2.5, and 4 dpi samples as early, middle, and late infection samples. For *Py. ultimum,* infections were initiated using a hyphal plug, and at 1.5 dpi the tuber was dissected into concentric zones representing early, middle, and late stages of colonization. The outer (early) zone included a 3-mm region comprised of one-quarter of nondiscolored tuber tissue and three-quarters reddish brown tissue. The middle region included 3-mm of brown tissue. The late sample included 3-mm of black tissue.

### Initial analysis of pathogen transcriptomes

RNA-seq was performed using the three infection stages described above, with three biological replicates. Early and late stages of growth of *Ph. infestans* and *Py. ultimum* on rye broth and pea broth were also analyzed to allow the identification of infection-induced genes. For *Ph. infestans*, the early and late media cultures represented non-sporulating and sporulating timepoints, respectively. With *Py. ultimum,* sporangia-like hyphal swellings were detected in the late cultures, but their density was <5% of that of *Ph. infestans* sporangia.

The number of reads from the tuber and media samples are shown in Additional file 1: Table S1. The fraction coming from the pathogens increased over the course of infection. In early, middle, and late stages of tubers infected with *Ph. infestans,* 3.9, 30, and 76% of reads mapped to the pathogen, respectively. This increased from an average of 13 million (MM) to 234 MM reads for each early and late tuber replicate, respectively. In tubers infected with *Py. ultimum* the early, middle, and late samples resulted in 24, 88, and 90% of reads mapping to the pathogen, increasing from an average of 38 MM to 74 MM per replicate. The mapping percentage from late *Py. ultimum*-infected tubers is close to that of pure cultures (90 vs. 93%), indicating that little potato RNA persists late in infection. In contrast, host transcripts were eliminated more slowly with *Ph. infestans.* Using a minimum CPM (counts per million mapped reads) cut-off of 1.0, 14,081 *Ph. infestans* and 12,164 *Py. ultimum* genes were expressed in at least one media or tuber condition. CPM calls for both species are shown in Additional file 2: Table S2, along with annotations for each gene.

To confirm that *Ph. infestans* went through a normal disease cycle, four stage-specific genes were examined (Fig. 1c). Effector Avr3a and haustorial protein Hmp1 mark the biotrophic stage of infection [22]. Both of their genes were expressed at much higher levels in the early and middle stages of tuber colonization than the late stage. Induced more than 100-fold in the late sample was the gene encoding a marker for necrotrophy, NPP1 [22]. The same was observed for the gene encoding centrin, which is a marker for sporulation, which occurs at the end of the disease cycle [23].

To test further the quality of the data and start to obtain insight into pathogen biology, we performed principal component analysis (PCA). Tight clustering was seen for each biological replicate (Fig. 1d,e). Early rye and pea samples clustered in both species, but not with early tubers, indicating major divergence in the transcriptomes of “young” mycelia in tubers and artificial media.

Heatmaps based on hierarchical clustering also indicated that major changes occurred in both pathogens during *ex planta* and *in planta* growth (Fig. 1b). Based on a fold-change ratio threshold of 4.0 and a false discovery rate (FDR) cut-off of 0.05, 15.5% and 10.0% of *Ph. infestans* and *Py. ultimum* genes were expressed differentially, respectively, in early tubers compared to early media (Fig. 1f). This indicated that while transcriptomic reprogramming occurred in both pathogens, a greater fraction of genes in *Py. ultimum* were expressed constitutively. One type of gene that underlies this difference are RXLR effectors, which are numerous and often infection-induced in *Ph. infestans* but absent from *Py. ultimum*.

Another difference between the species was observed when comparing early and late tubers. While only 9.4% of *Py. ultimum* genes changed by ≥4-fold (FDR < 0.05) between early and late infection, 45% of *Ph. infestans* genes changed to the same degree (Fig. 1f). One explanation for this disparity is that during late infection *Ph. infestans* switches from biotrophic to necrotrophic growth, and sporulates.

### Gene ontology (GO) analysis

**Fig. 2:**
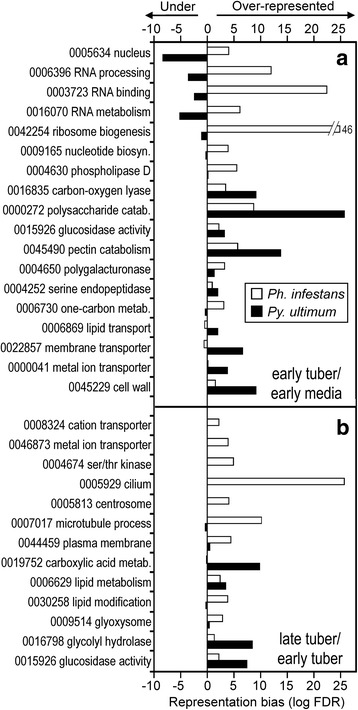
Gene ontology (GO) enrichment analysis. Indicated are terms that are over- or under-represented in genes of *Ph. infestans* (white bars) or *Py. ultimum* (black bars) that are up-regulated in **a** early tuber compared to the mean of early rye and pea media, and **b** late tuber versus early tuber

Infection-induced GO categories (Fig. 2) that were over-represented in both species included carbon oxygen lyase, serine endopeptidase, polysaccharide metabolism, and pectin catabolism; the latter categories represent cell-wall degrading enzymes (CWDEs)*.* This likely reflects the fact that *Ph. infestans* grows biotrophically and causes minimal damage to the host, while *Py. ultimum* is a necrotroph. Many changes observed for *Ph. infestans* resembled those described in a microarray study of *Ph. capsici*, such as the up-regulation early in infection of RXLR effectors and genes involved in transcription [22].

In comparisons of early tubers with media, differences between the pathogens included the RNA metabolism, RNA binding, and ribosome biogenesis categories. These GO terms were over-represented in *Ph. infestans* but under-represented in *Py. ultimum* (Fig. 2a). GO categories overrepresented in *Py. ultimum* but not *Ph. infestans* included those associated with transmembrane transporters. As will be described later, many ABC transporters are expressed highly during tuber colonization by *Py. ultimum*. A similar pattern was reported for necrotrophic fungal plant pathogens [1].

A few over-represented GO terms were shared by the two species when comparing late to early tubers (Fig. 2b). These included glycosyl hydrolases, glucosidases, and enzymes associated with lipid metabolism, which would generate assimilable carbon for the pathogen. This may reflect a subtle change from earlier stages, when damage to host structures may be used more to allow hyphae to penetrate host cells than to liberate metabolizable carbon. Most over-represented GO terms were however unique to *Ph. infestans.* These included categories associated with structural components of sporangia (e.g. centrosome, cilium) and signal transduction (serine/threonine kinase). The latter could participate in spore-associated functions or a transition to necrotrophic growth*.* A prior study of *Ph. infestans* observed changes in many protein kinases during sporulation [24]. While older cultures of *Py. ultimum* form hyphal swellings and sexual spores, their concentrations were low and likely insufficient to cause obvious gene expression changes.

Several genomic processes may explain the evolution of the differences between *Ph. infestans* and *Py. ultimum* described above and later in this paper. Expression patterns may have changed due to mutations in promoters or transcription factors. Other possibilities are gene gain or loss, including the expansion or contraction of families. In the following sections, we address these processes as well as factors that may underlie biotrophic and necrotrophic growth. First, we will focus on functional classes of genes implicated in pathogenesis. Then, we will study ortholog expression patterns in order to address regulatory subfunctionalization genome-wide.

### Expression of putative pathogenicity genes

**Fig. 3:**
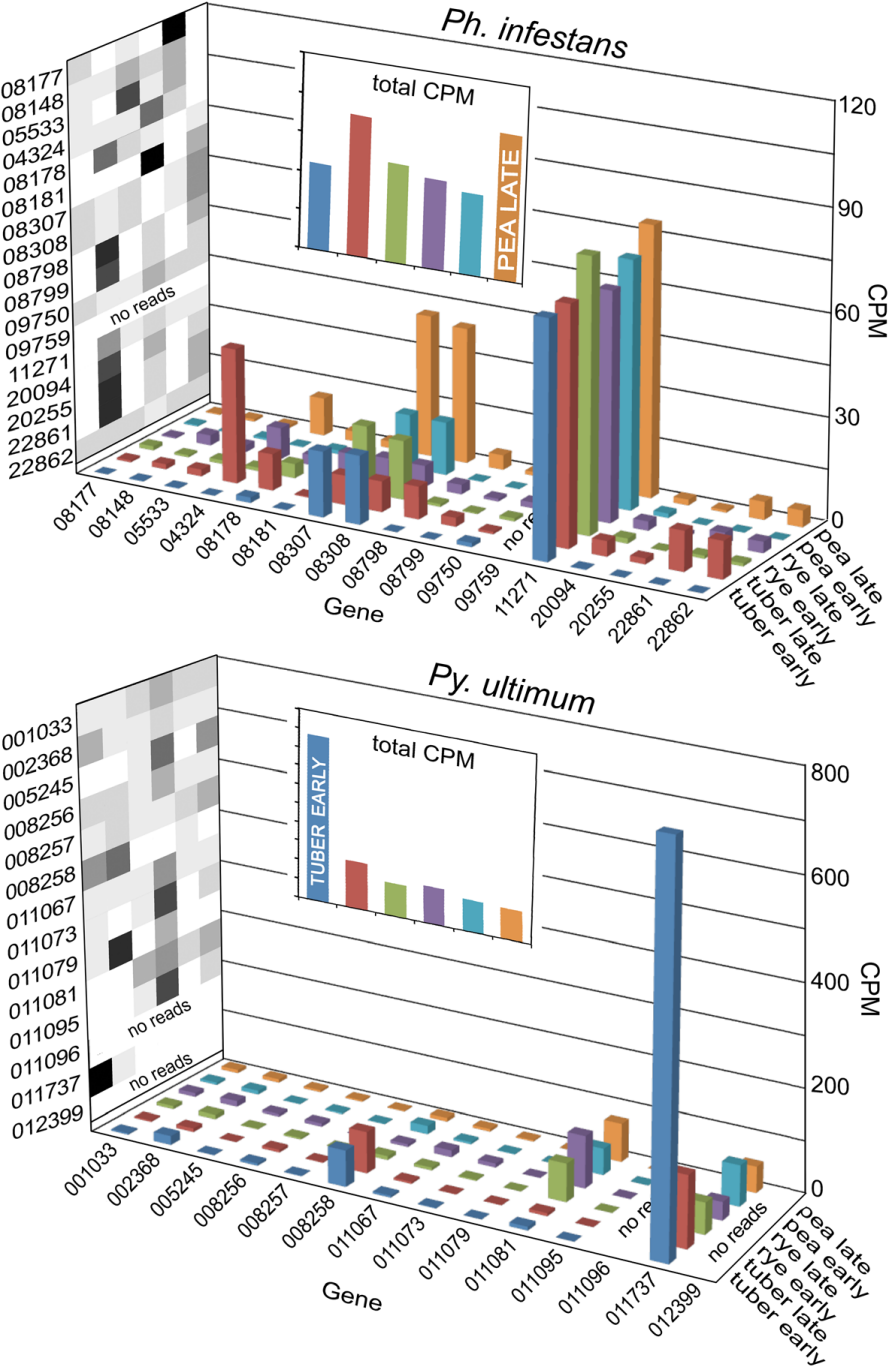
Expression of polyphenol oxidases. The colored bar graphs in the bottom plane show CPM values for individual genes in the six growth conditions in *Ph. infestans* (top) and *Py. ultimum* (bottom). Using the same color scheme, the bar graphs on the back wall portray aggregate CPM for all genes; those with the highest levels (pea late and tuber early in the upper and lower graphs, respectively) are indicated. Heatmaps based on per gene-normalized values are shown in grey scale on the left wall. Genes with CPM <1 are shown as “no reads”

Preliminary explorations indicated that transcription should be addressed both as the expression patterns of individual genes and aggregate (summed) CPM values within functional categories. To explain why, Fig. 3 illustrates a family of polyphenol oxidases, which may help protect pathogens against toxins or stress [25]. These have 17 paralogs in *Ph. infestans* and 14 in *Py. ultimum*. Conventional heatmap analysis using per-gene normalized data (black and white checkerboards in Fig. 3) indicates that most of the genes are expressed in both species at their highest levels in late tuber and late rye, respectively. However, this is a distorted view since some genes are expressed more highly than others. When the expression of all genes are considered by adding together their individual CPMs, we observe that transcript levels in *Py. ultimum* are highest in early tuber, and fairly constant in *Ph. infestans*.

Similar patterns were seen within many gene families, which based on OrthoMCL analysis encompass about half of the genes in the two species. Therefore, expression will be described in the following sections both as aggregate CPM and in heatmaps as per-gene normalized values. The latter is the most common way of illustrating expression data, but masks the divergence in CPM of individual genes. It is acknowledged that weakly-expressed genes may have important functions, and that mRNA and protein levels may not increase proportionally.

#### Secreted proteases

These may degrade structural components of the host to facilitate pathogen ingress, liberate nutrients, or defeat host defense proteins [26]. We predict that 29 of 101 *Ph. infestans* and 72 of 171 *Py. ultimum* proteases, respectively, are secreted. These numbers are larger than those reported previously [17] since our study included more families of proteases. Of the genes encoding secreted proteases, 25 and 64 were expressed in at least one tissue of each species, respectively, based on a CPM cut-off of 1.0.

**Fig. 4:**
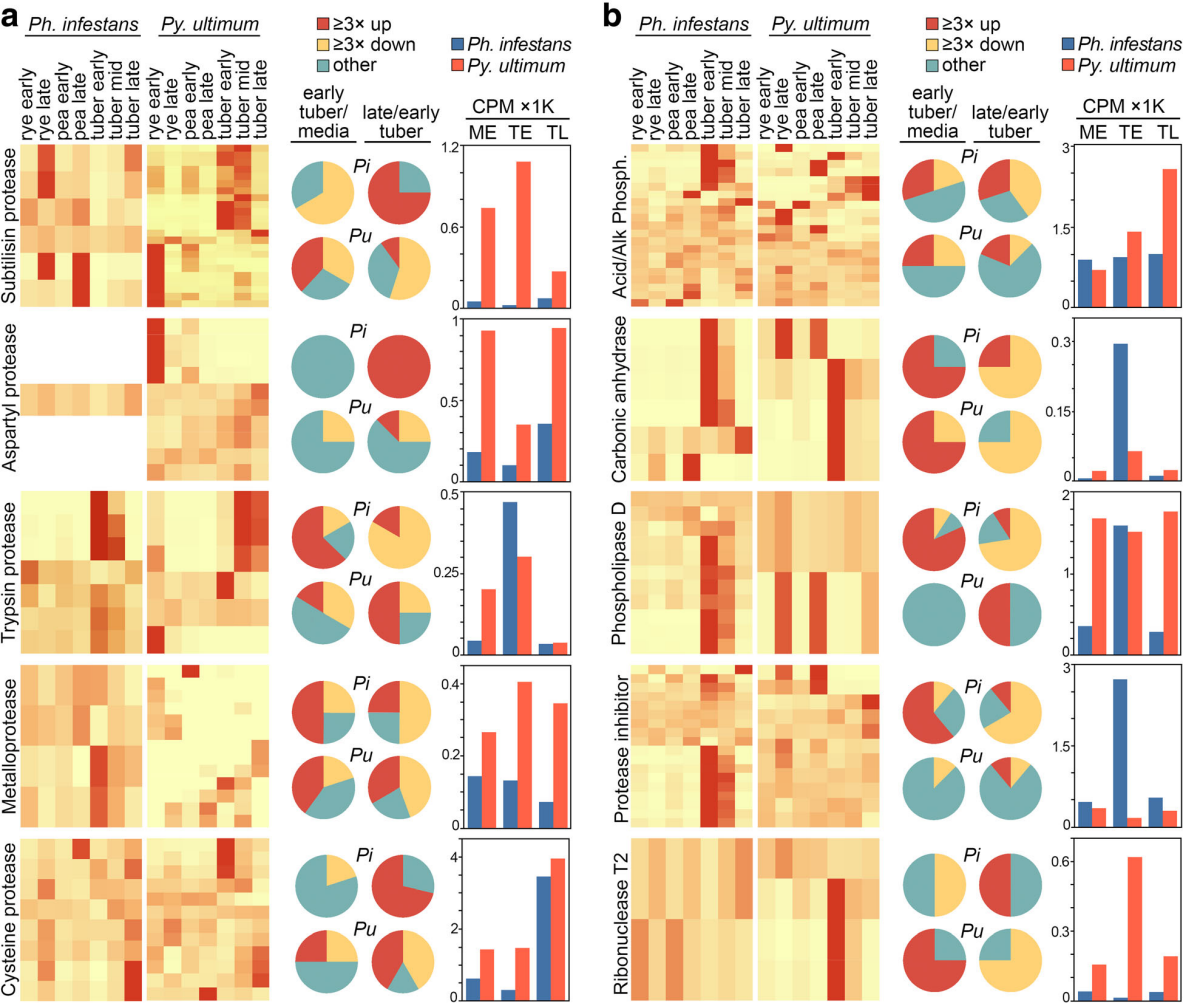
Expression of selected pathogenesis-related genes. Illustrated are **a** secreted proteases and **b** five other classes of secreted pathogenicity factors. Shown on the left side of each panel are heatmaps of genes from *Ph. infestans* (left) and *Py. ultimum* (right) that have CPM ≥1 in at least one growth condition. The pie charts in the middle of each panel represent the fraction of genes from *Ph. infestans* (Pi) and *Py. ultimum* (Pu) that are up-regulated by ≥3-fold (red) or down-regulated (yellow) in early tuber compared to media, or late tuber compared to early tuber. Genes that show smaller changes are represented by the teal (blue-green) slice. Shown on the right side of each panel are bar charts indicating the aggregate CPM of all genes in each functional group in early rye and pea media (averaged; ME), early tuber (TE), and late tuber (TL). Note that *Ph. infestans* expresses a single aspartyl protease

Distinct trends were observed for each family (Fig. 4a). In this and subsequent figures, the left column is a heatmap based on per-gene normalized values; only genes with CPM ≥1 in at least one growth condition are included. The pie charts in the center denote the fraction of genes that are up- and down-regulated by ≥3-fold in early tuber versus early media (average of rye and pea), and late tuber versus early tuber. The right column displays the aggregate CPM of the genes in early media, early tuber, and late tuber.

Several classes of proteases showed consistently higher transcription, and more infection-induced genes, in *Py. ultimum* compared to *Ph. infestans.* Eight of 23 expressed (e.g. having CPM ≥1) subtilisin proteases in *Py. ultimum* were induced in early tubers compared to none of the six expressed *Ph. infestans* genes*.* Combined with expansion of the family in *Py. ultimum*, this resulted in 50-fold higher CPM in that species compared to *Ph. infestans.* mRNA levels were also higher in *Py. ultimum* for aspartyl proteases, which were expressed from one and ten genes in *Ph. infestans* and *Py. ultimum*, respectively. Metalloproteases also had higher total CPM in *Py. ultimum* at each stage. Although more metalloproteases were tuber-induced in *Ph. infestans,* due to low expression this did not translate into higher overall CPM.

Unlike the enzymes mentioned above, trypsin proteases in *Ph. infestans* and *Py. ultimum* had similar numbers of expressed genes (seven and six, respectively) and aggregate CPM in early tubers. More genes were induced in early tubers compared to media in *Ph. infestans* than *Py. ultimum* (four and one, respectively). CPM in both species dropped by >10-fold in late tubers. While half of the *Py. ultimum* genes were late-induced*,* these were expressed at low levels and did not prevent the decline in total CPM.

While levels of subtilisin, trypsin, and metalloproteases all fell in late versus early tubers, the opposite pattern was seen for the eight and 12 cysteine proteases expressed by *Ph. infestans* and *Py. ultimum*, respectively. Both species had genes induced in late tuber, which caused aggregate CPM to rise. In late tubers, cysteine proteases were expressed at about 4000 CPM in both species, which was three to seven-fold more than the other proteases combined.

Overall, the data suggest that proteases act in two phases during infection. The initial phase may involve trypsin proteases in *Ph. infestans* and subtilisin, metallo-, and trypsin proteases in *Py. ultimum*. The subtilisin and metalloproteases are known for their abilities to function in inhospitable environments, which may include the apoplast [27]. A second wave in both pathogens may involve cysteine and aspartyl proteases. The broader arsenal of proteases secreted by *Py. ultimum* may contribute to its ability to infect multiple hosts.

#### Secreted phosphatases

Most secreted phosphatases act on organic compounds to release soluble phosphate, which can be assimilated by the pathogens [28]. *Ph. infestans* and *Py. ultimum* are predicted to encode 26 and 21 of these enzymes, of which 21 and 20 were expressed at CPM ≥1, respectively (Fig. 4b). Of the expressed genes, two per species encoded acid phosphatases with the remainder encoding alkaline-favoring enzymes.

In early tubers compared to media, six acid and zero alkaline phosphatases were induced in *Ph. infestans,* compared to two and two, respectively, in *Py. ultimum.* Both species had a few late infection-induced phosphatases, and a similar fraction of late-repressed genes. As a consequence, aggregate CPM values of the *Ph. infestans* enzymes stayed fairly constant during tuber infection at about 1000 CPM, with an approximate 4:1 ratio of acid to alkaline phosphatases. The predominance of acid-favoring enzymes may be explained by the fact that leaf apoplasts and tubers are mildly acidic [29].

The situation in *Py. ultimum* was quite different. Levels of acid phosphatase in *Py. ultimum* showed only minor changes between growth conditions, staying near 850 CPM. However, its alkaline phosphatases went from <1 CPM in media to 358 in early tuber and 1648 in late tuber. As a consequence, there was a shift from an excess of acid phosphatases in media and early tubers to an excess of alkaline activity in late tubers. This was due largely to PYU1_G001049, which accounted for 96% of total phosphatase CPM in late tubers. The shift might be explained by a need for *Py. ultimum* to prepare for growth in soil after the nutrients from its plant host become depleted. In alkaline soils, inorganic phosphate tends to form insoluble compounds, so alkaline phosphatase may help liberating phosphate from organics to support growth.

#### Secreted carbonic anhydrase

This converts carbon dioxide to bicarbonate, generating a proton which may contribute to pH homeostasis. The reaction also forms bicarbonate, which may be a co-factor or co-substrate in fatty acid and cAMP pathways [30]. Prior studies indicated that some carbonic anhydrases are infection-induced in *Ph. infestans* [20, 31].

*Ph. infestans* and *Py. ultimum* are predicted to encode 16 and eight carbonic anhydrases, of which seven and eight were predicted to be secreted and six and four expressed at CPM ≥1, respectively (Fig. 4b). A majority were up-regulated in early tubers compared to media. There is a corresponding increase in aggregate CPM in early tubers, but more in *Ph. infestans* (64-fold) than *Py. ultimum* (three-fold). The aggregate CPM in also much higher in *Ph. infestans* in early infection*.* This may reflect a role in maintaining pH levels optimal for other secreted enzymes during biotrophy. In both species, a role in balancing pH may also be indicated by the induction of several carbonic anhydrase genes during late growth in media, and in late tubers in *Ph. infestans.* Interestingly, this involved genes other than those induced during early infection.

#### Secreted phospholipase D

A novel feature of certain oomycetes are secreted forms of phospholipase D, which in addition to degrading structural phospholipids can generate the signaling molecule phosphatidic acid [31, 32]. Whether the extracellular generation of this signaling compound affects the pathogen or host is unknown. *Ph. infestans and Py. ultimum* encode 11 and three of the secreted forms of these enzymes, of which 11 and two were expressed at CPM ≥1, respectively (Fig. 4b).

More than three-quarters of the *Ph. infestans* genes, but no *Py. ultimum* genes, were induced in early tubers. This is consistent with a prior report that some of the former were induced during leaf infection [20]. In our study, expression of nearly all *Ph. infestans* genes declined towards the end of the biotrophic stage. However, secreted phospholipases do not seem to define biotrophic growth since both species had similar CPM levels, and the highest levels, in early tubers.

#### Secreted protease inhibitors

Members of the Kazal and cysteine protease inhibitor families help protect *Ph. infestans* against host enzymes [33]. The transcription patterns and functions of such proteins in necrotrophic oomycetes are not described. *Ph. infestans and Py. ultimum* encode 36 and 19 such proteins, respectively, of which 28 and 18 are predicted to be secreted and 18 and 11 expressed at CPM ≥1, respectively (Fig. 4b). About two-thirds of the *Ph. infestans* genes were induced in early tubers compared to media, declining to low levels late in infection. In contrast, nearly all of the *Py. ultimum* genes were expressed constitutively. Two were induced ≥3-fold in late tubers, but contributed little to total CPM.

#### Secreted ribonuclease T2

*Ph. infestans* and *Py. ultimum* encode two and six secreted forms, respectively, of this non-specific ribonuclease, with two and four expressed at CPM ≥1 (Fig. 4b). In *Ph. infestans*, one of the genes was up-regulated during late infection, as well as in late rye and pea cultures. In *Py. ultimum,* three genes were expressed at the highest level in early infection, resulting in peak aggregate CPM at that stage. Of the putative pathogenicity factors addressed by this study, this was one of the few that appeared to be transcribed at a higher level during late infection by *Ph. infestans,* and could thus be linked to necrotrophic growth in both species.

In animals, a portion of secreted T2 enzymes are retained within the cell. There are also examples of secreted T2 enzymes being taken up by animal or plant cells [34]. Therefore, the oomycete enzymes might be used to scavenge extracellular nucleic acids for nutrients, turn over pathogen mRNA, or modulate host defenses.

#### NPP family

Certain oomycetes, fungi, and bacteria express this family of proteins, which cause necrosis in plants. NPP1, encoded by gene PITG_16866 of *Ph. infestans*, is often used to mark late infection (Fig. 1c). It is known, however, that *Phytophthora* spp. encode multiple NPP proteins. Many are neither expressed late in infection nor cause necrosis and have unknown cellular roles [35, 36].

**Fig. 5:**
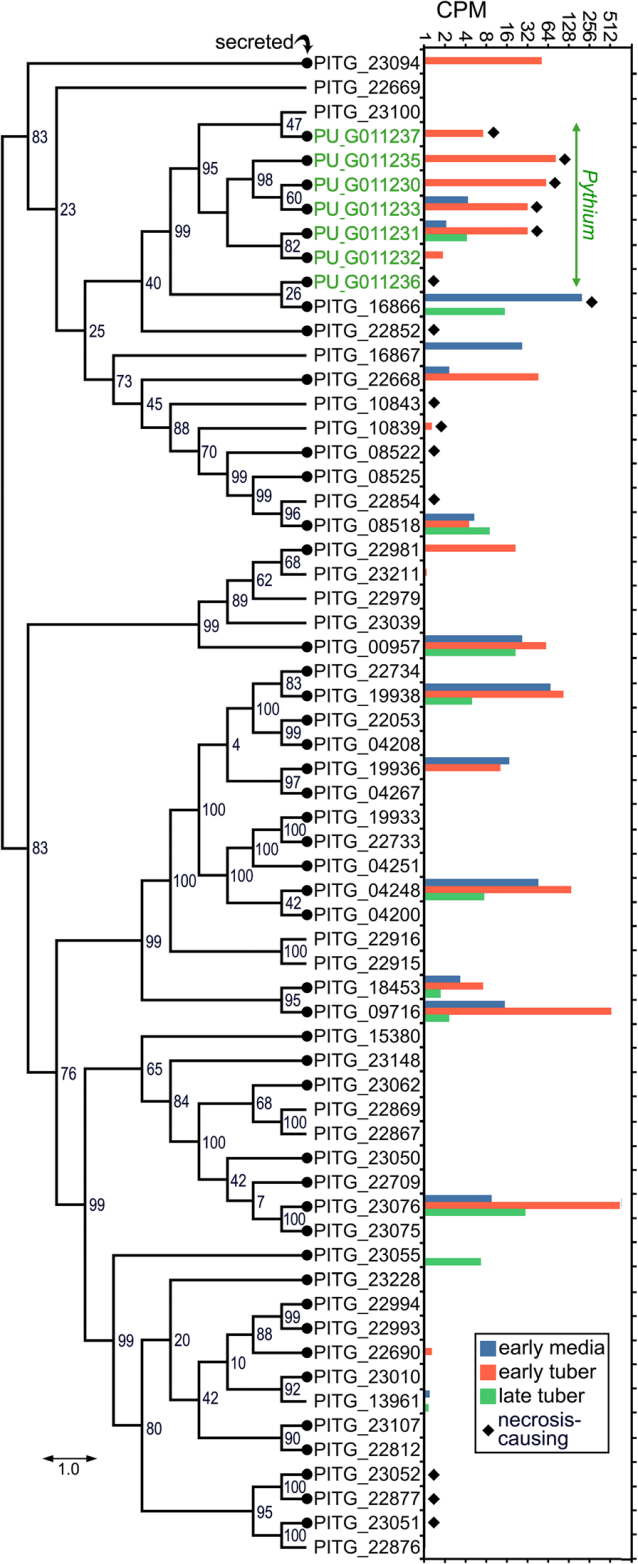
Structure and expression of NPP (NLP) family. The left side of the figure is a cladogram of the genes from *Ph. infestans* (PITG prefix, black lettering) and *Py. ultimum* (PU prefix, green lettering). Bootstrap numbers from PhyML are shown at nodes. The right side of the figure indicates the CPM values in early media (average of early rye and pea, green), early tubers (blue) and late tubers (red bars). An absence of bars indicates CPM ≤1.0. Proteins predicted to be secreted are marked by black circle on the terminal branches of the tree, and those bearing the residues required for plant necrosis are marked by a diamond. These correspond to amino acids D112, H120, D123, and E125 of *Phytophthora capsici* NLP1 [36]. While PITG_22668 contains those residues, it also contains a S113E substitution which was associated with low necrosis in *Ph. capsici*

*Ph. infestans* encodes 55 NPP proteins, of which 40 are predicted to be secreted. Twenty-two were expressed in at least one growth condition at CPM ≥1. Of seven *NPP* genes in *Py. ultimum,* six were expressed and all encoded secreted proteins (Fig. 5). Six *Py. ultimum* genes, but only nine *Ph. infestans* genes, were conserved at residues shown to be essential for inducing necrosis [36].

Intriguingly, transcription of the family varied substantially between the species. All of the *Py. ultimum* genes were expressed much more in early than late tubers. In *Ph. infestans,* three genes including PITG_16866 were expressed ≥3-fold higher in late compared to early tubers, while three other genes were ≥3-fold higher in early tubers. Notably, the *Ph. infestans* genes expressed in early tubers all lacked the residues needed for inducing necrosis. PITG_16866 was expressed at high levels in media and late tubers, but not early tubers, suggesting that its expression is suppressed during early infection to help maintain biotrophy. The proteins predicted as necrosis-causing had an aggregate CPM in early and late tubers of 1.9 and 16 in *Ph. infestans,* and 213 and 6.5 in *Py. ultimum*, respectively, consistent with their association with the necrotrophic stage.

Phylogenetic analyses indicated that the *Py. ultimum* genes form a well-supported clade with PITG_16866*,* even though they have opposite stage-specific expression patterns (Fig. 5). Despite extensive expansion of the family in *Ph. infestans,* the advantages of biotrophy to that species in early infection appears to have selected for mutations of the residues required for plant necrosis, in addition to remodeling its transcription pattern relative to that of *Py. ultimum.*

#### CRN family

This includes effectors that are secreted and translocated into plant nuclei [37]. However, most members of the family are not secreted and play unknown roles. Like *NPP1,* the *CRN* family is expanded in *Ph. infestans* relative to *Py. ultimum* with 196 and 26 members, respectively [17]. Of these, expression was detected at CPM ≥1 for 132 and 14 genes, respectively. No *Py. ultimum* CRN genes were infection-induced. Ten *Ph. infestans* genes (PITG_04767, 04769, 05039, 16,575, 16,627, 17,540, 18,835, 19,340, 21,058, 22,536) had ≥3-fold higher expression in tubers compared to media, although only four of those encode secreted proteins. That most CRNs are not induced *in planta* has been described [18].

#### RXLR family

Like CRNs, these host-translocated effectors are known for playing roles in suppressing plant immune responses [38]. They have members in *Ph. infestans* but not *Py. ultimum.* Studies in several species of *Phytophthora* have shown that different RXLRs show distinct patterns of transcription during the disease cycle [18, 22]. To understand this in more detail, we integrated the RNA-seq data from the current study with data from our recent study of *Ph. infestans* development, which was also performed with isolate 1306 [24]. Of the approximately 500 RXLRs annotated in the reference genome of *Ph. infestans,* 297 were expressed at CPM ≥1 in 1306. This may understate the fraction of expressed RXLRs due to variability within the family; some genes in the reference genome may not be represented within 1306, which may also contain novel RXLRs.

**Fig. 6:**
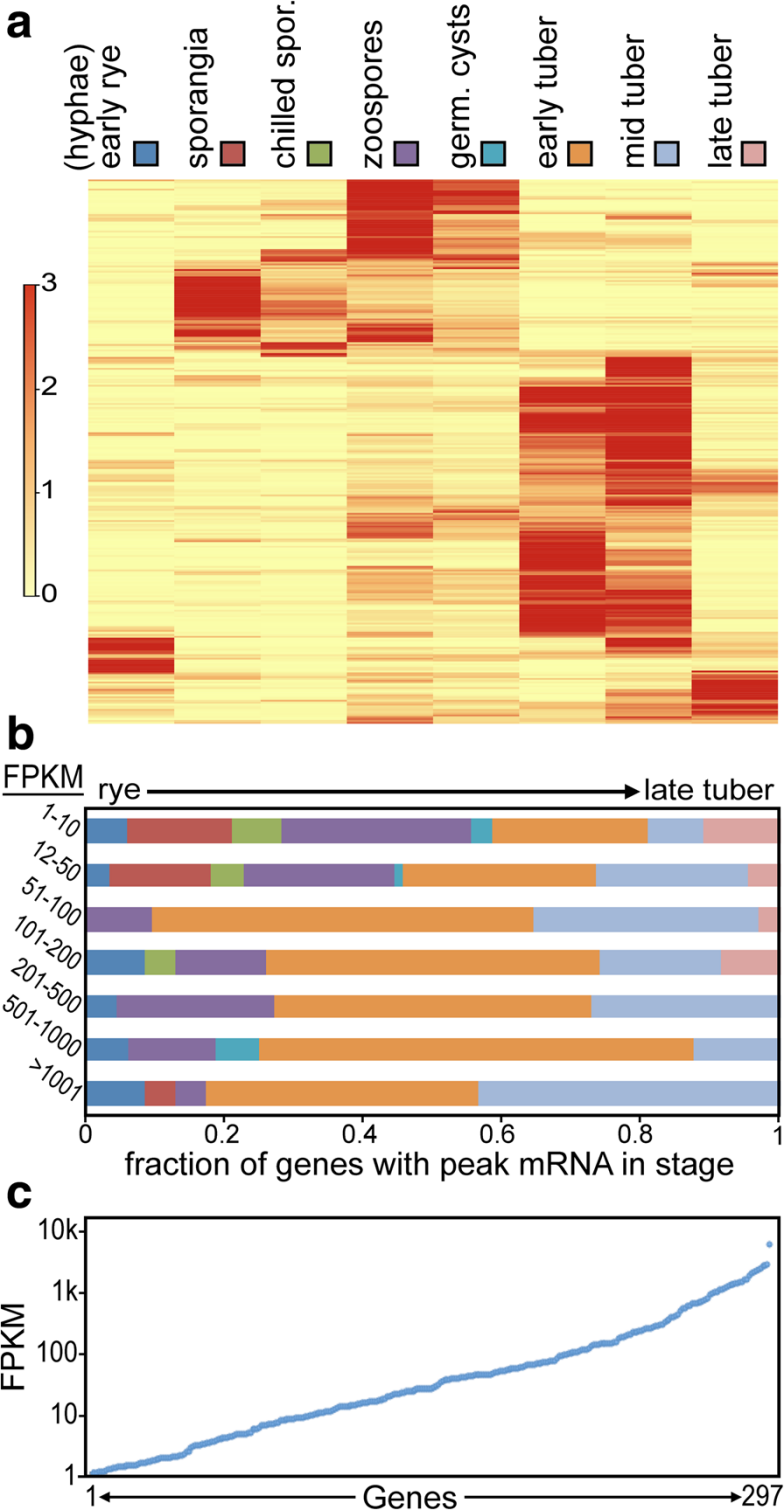
Expression of *Ph. infestans* RXLR genes. **a** heatmap of mRNA levels in nonsporulating hyphae from rye media (early rye), purified sporangia, sporangia chilled to initiate zoospore formation, swimming zoospores, cysts germinated in water, and tubers. Color codes for each sample are shown in boxes at the top of the panel; these correspond to the bar segments in panel b. **b** fraction of genes showing peak expression in the eight tissue samples, classified by peak FPKM values. The bar segments are presented in the same order (left to right) as the tissues in panel a. **c** maximum FPKM level of 297 individual RXLRs

As shown in the heatmap in Fig. 6a, only a few RXLRs were expressed in rye media, and most were induced in a preinfection spore stage or in tubers. About 10, 19, and 2% showed peak expression in sporangia, zoospores, and germinated cysts, respectively. The latter were made by placing cysts in water in plastic dishes, and it is possible that more are induced when cysts germinate on a host. The majority of RXLRs were induced *in planta,* with 34, 20, and 6% showing peak expression in early, middle, or late tubers, respectively.

Besides diversity in stage-specific transcription, mRNA levels varied widely within the family (Fig. 6c). Of genes deemed to be expressed, the median FPKM across all life stages was 25.0 compared to a median of 13.3 for all *Ph. infestans* genes. Having FPKM > 1000 in any one stage were 23 genes, with the highest (PITG_23131) peaking in middle tubers with a FPKM of 5762. The RXLRs showing medium-to-high FPKM were most likely to be expressed maximally in early or middle tubers (Fig. 6b). More weakly expressed RXLRs, i.e. those with FPKM between 1 and 50, were more likely to reach peak expression in zoospores or sporangia. RXLRs expressed at their highest levels in late tubers showed moderate expression compared to those expressed in early and middle tuber timepoints.

### Plant cell wall degrading enzymes (CWDEs)

Oomycetes and fungi secrete diverse enzymes to depolymerize the cell walls of their hosts [19, 39–43]. Since both oomycete and plant walls are comprised mostly of β-1,3 and β-1,4 glucans, they are affected by similar enzymes. The presence of transmembrane domains and glycosylphosphatidylinositol (GPI) anchors would be likely features of enzymes that remodel oomycete walls during hyphal extension. Therefore, to focus on plant-targeted CWDEs, the following analyses are restricted to secreted proteins that lack transmembrane domains and GPI anchors.

#### β-1,3-glucanases

**Fig. 7:**
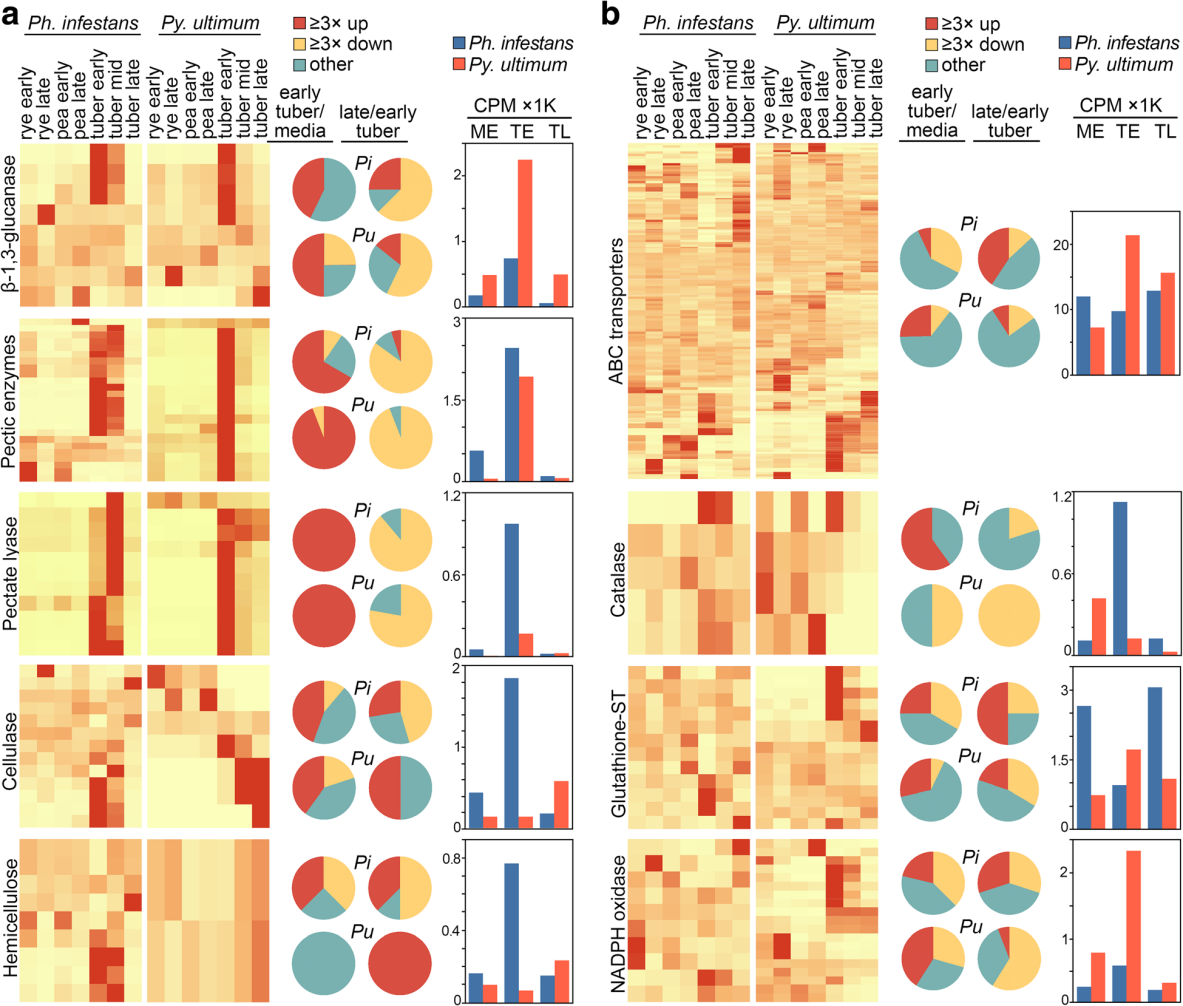
Expression of selected pathogenesis-related genes. Illustrated are **a** cell wall-degrading enzymes (CWDEs) grouped by activity, and **b** genes potentially involved in detoxification. The format of the figure is the same as in Fig. 4

These are targeted by glycoside hydrolase families GH17 (β-1,3-glucosidase) and GH81 (β-1,3-glucanase). *Ph. infestans* and *Py. ultimum* are predicted to encode three and five extracellular GH17 enzymes, and five and three GH81 enzymes, respectively. All were expressed based on the CPM threshold of 1.0 (Fig. 7a).

About 80 and 42% of the genes were infection-induced in *Py. ultimum* and *Ph. infestans*, respectively, with the highest levels observed for both species in early tubers. Total CPM was three and ten-fold higher in *Py. ultimum* compared to *Ph. infestans* in early and late tubers, respectively. One *Py. ultimum* gene, PYU1_G013792, was expressed at very high levels, peaking in tubers at 1776 CPM or 79% of total β-1,3-glucanase CPM. In contrast, no single *Ph. infestans* gene accounted for more than 20% of total CPM.

Infection-induced enzymes from *Ph. parasitica* that target β-1,3-glucans have been identified in its interaction with lupin [41]. Such enzymes may be used to degrade plant cell walls or callose, which plants deposit in response to pathogens [44]. Both roles are consistent with the higher expression of the enzymes in *Py. ultimum,* in which the need to disrupt cell walls may be more important and the ability to repress host defenses more limited than in *Ph. infestans*.

#### Cellulases

This activity is conferred primarily by β-glucosidase (GH1, GH3), endo-β-1,4-glucanase (GH5, GH6), and exo-β-1,4-glucanase (GH7) [41]. *Ph. infestans* and *Py. ultimum* are predicted to encode one and zero GH1 proteins, respectively, of which the former was expressed at CPM ≥1 in at least one growth condition; eight and three GH3 proteins, of which seven and three were expressed; zero and four GH5 enzymes, with three of the latter expressed; four and zero GH6, all of the former expressed; and one and one GH7, both expressed (Fig. 7a).

Patterns of expression between the species were very different. In early infection, total cellulase CPM was more than ten-fold higher in *Ph. infestans* than *Py. ultimum,* due largely to increased expression of enzymes in categories GH3 (PITG_17546, PITG_22095), GH6 (PITG_18388), and GH7 (PITG_06788). In contrast, in late tubers, CPM was three-fold higher in *Py. ultimum* than *Ph. infestans*. The increase in *Py. ultimum* was due mostly to two highly-expressed GH3 (PYU1_G000955, G000957) and one GH5 genes (PYU1_G012486). Although total *Ph. infestans* cellulase CPM fell nine-fold in late tubers, one GH1 (PITG_01399) and two GH3 genes (PITG_15905, 03140) were up-regulated.

#### Hemicellulases

To fully degrade the cell wall, pathogens must digest the heteropolymeric matrix that coexists with cellulose. Enzymes participating in this include β-mannosidase (GH2), endo-1,4-β-xylosidase (GH3), endo-1,4-mannosidase (GH5), β-1,4-xylanase (GH10), and xyloglucan specific-endo-β-1,4-glucanase (GH12).

*Ph. infestans* encodes a plethora of these enzymes. This includes three GH3 proteins, of which two were expressed at CPM ≥1; one GH5 enzyme, which was expressed; two GH10s, both expressed; and six GH12s, of which four were expressed. Nearly half were induced strongly in early tubers compared to media, paralleling the rise in cellulases. As observed in many other functional categories, a majority (61%) of hemicellulase CPM came from a single GH12 gene, PITG_08944.

In contrast to *Ph. infestans, Py. ultimum* encodes only one GH2 and one GH3, both of which were expressed. Unlike the *Ph. infestans* enzymes, none were infection-induced, and their CPM in early tuber was only one-tenth that of *Ph. infestans.* Expression increased four-fold in late tubers, achieving levels similar to those of *Ph. infestans*.

#### Pectic enzymes

Homogalacturonan, rhamnogalacturonan-I, and substituted galacturonans comprise the pectin matrix of plant cell walls [40, 41]. Enzymes degrading these include pectin lyase (PL1), pectate lyase (PL3), rhamnogalacturonan lyase (PL4), polygalacturonase (GH28), α-rhamnosidase (GH78), rhamnogalacturonyl hydrolase (GH105), and α-L-arabinofuranosidase (GH43). Their different specificities may control the overall rate of pectin digestion, by affecting the hydrolysis of its side chains.

Each pectin-degrading activity is encoded by multigene families in *Ph. infestans.* Seven of 12 predicted PL1 genes were expressed, as were 11 of the 16 PL3, one of two PL4, all 14 GH28, one of two GH78, both GH105, and none of the GH43 genes. Two-thirds were induced during early tubers, causing a six-fold increase in total pectic enzyme CPM compared to media. All genes declined in late tubers, reducing aggregate CPM by 32-fold. The pectin-degrading activities of *Py. ultimum* include 12 PL1s, all of which were expressed; 14 PL3s, of which 10 were expressed; five GH28s, with three expressed; and two PL4s, both expressed. Unlike *Ph. infestans,* there were no GH78 or GH105 genes [40] but two GH43 genes, both of which were expressed. All of the *Py. ultimum* genes were up-regulated significantly in early tubers, and declined in late tubers.

In most cases, there was a good correlation between gene number and aggregate CPM. For example, the three and 14 GH28 enzymes expressed in *Py. ultimum* and *Ph. infestans,* respectively, yielded 177 and 1375 CPM in early tubers. Similarly, the expansion of expressed, secreted PL1 genes in *Py. ultimum* versus *Ph. infestans* to 12 from seven resulted in CPM totals of 1613 and 633, respectively. However, this proportionality was not observed for PL3, where 10 and 11 genes in *Py. ultimum* and *Ph. infestans* resulted in CPM values of 164 and 992, respectively. Contributing to this disparity was one highly expressed and tuber-induced *Ph. infestans* gene, PITG_14168, which yielded 810 CPM in early tubers.

*Ph. infestans* also encodes six secreted pectinesterases (CE8), all of which were expressed. Their expression patterns are not included in Fig. 7 since *Py. ultimum* is predicted to lack this activity [40]. The *Ph. infestans* genes were each induced in early tubers, with aggregate CPM values of 139, 1277, and 40 in media, early tubers, and late tubers, respectively. Most were shown to be up-regulated in zoospores and germinated cysts compared to media, suggesting that they anticipate plant infection [24].

Pectin-degrading enzymes are of particular interest when comparing the outcomes of infection, since while potatoes remain quite firm at the conclusion of the *Ph. infestans* disease cycle, the tubers are liquefied by *Py. ultimum*. Since pectin and pectate lyases (PL1) and polygalacturonase (GH28) were shown to be the most effective in macerating tubers [45], it was surprising to find that the aggregate CPM of PL1 was about three-fold higher in *Ph. infestans* in early tubers, and GH28 CPM was eight-fold higher in *Ph. infestans.* A related enigma is why *Py. ultimum* does not express any pectin methylesterases; these remove methyl groups as a first step towards exposing the polymer to other enzymes, which would facilitate liquefaction of the tuber.

Besides the enzymes described above, *Ph. infestans* and *Py. ultimum* are predicted to encode one and two GH43 arabinofuranosidases, respectively, of which only the *Py. ultimum* enzymes were expressed. These also target pectin, although hemicellulose and arabinogalactans may also be substrates. Their aggregate CPM in *Py. ultimum* rose from 1.1 to 59 in media and early tubers, respectively, before falling to 6.0 in late tubers.

### Genes involved in secretion

Because most pathogenicity factors are extracellular, we examined three classes of proteins involved in the classic secretory pathway. These were SNARE proteins which mediate vesicle fusion, Rab GTPase proteins which help regulate vesicle fusion, and orthologs of other yeast genes involved in trafficking vesicles for secretion [46].

Only a few genes showed strong differential expression in *Ph. infestans* or *Py. ultimum*, and there was only minor variation between the species in aggregate CPM (Additional file 3: Figure S1). One subtle difference was that the total CPM of genes encoding SNARE proteins was two-fold higher during tuber infection by *Py. ultimum* compared to *Ph. infestans.* While some genes encoding SNARE or Rab GTPases in *Ph. infestans* were up-regulated during infection (e.g. PITG_01853, 10,870, 18,718), their expression was not high enough to cause a major change in aggregate CPM. A difference was observed during early infection in the aggregate CPM of orthologs of yeast secretory genes, as this was 2.5 times higher in *Ph. infestans* than *Py. ultimum*. This was, however, attributable to just two highly expressed genes encoding chaperones.

It thus appears that most genes involved in secretion are expressed constitutively. It is however possible that some proteins bypass the classical trafficking pathway. This might involve a Golgi bypass, as suggested for animal cells [47].

### Detoxification pathways

#### ABC transporters

These efflux proteins may help defend the pathogens from environmental or plant-generated toxins [48]. *Ph. infestans* and *Py. ultimum* are predicted to encode 198 and 163 of these transporters, respectively, of which 153 and 148 were expressed at CPM ≥1 in least one tissue (Fig. 7b). More genes were induced in early tubers compared to media in *Py. ultimum* than *Ph. infestans* (34 and nine, respectively). In contrast, more were up-regulated in early versus late infection in *Ph. infestans* than *Py. ultimum* (59 and 12, respectively). Based on aggregate CPM, the *Py. ultimum* mRNAs were more abundant in early versus late infection, while the opposite trend was seen in *Ph. infestans.* Despite having 18% fewer ABC transporter genes, total ABC transporter CPM in *Py. ultimum* was from 25 to 220% higher than *Ph. infestans* in the tissues, with the largest difference in early tubers.

These results suggest that ABC transporters may play more significant roles in necrotrophic growth. This may be due to a need to eliminate host toxins, which as illustrated in Fig. 1a is likely to be more severe and occur earlier in potato leak than late blight*.* The up-regulation of ABC transporters in *Ph. infestans* in late tubers is consistent with a shift from biotrophic to necrotrophic growth, but could also be associated with sporulation*.* An earlier study indicated that about 10% of *Ph. infestans* ABC transporters were up-regulated in sporangia produced on artificial media [24].

#### Catalases

These may aid pathogens by eliminating H_2_O_2_ made by the host, or by delivering peroxide to the plant [49, 50]. *Ph. infestans* and *Py. ultimum* are predicted to encode five and six catalases, respectively, of which five and four were expressed in least one tissue with CPM ≥1.0 (Fig. 7b). Their patterns of expression were opposite of those of ABC transporters, as only *Ph. infestans* genes were significantly up-regulated in early tubers versus media. In addition, total catalase CPM was about ten-fold higher in *Ph. infestans* compared to *Py. ultimum* at all tuber stages*.* This may suggest that peroxide elimination is more critical for *Ph. infestans.*

Interestingly, 89% of catalase mRNA in *Ph. infestans* in tubers was from a single gene, PITG_07143, which along with PITG_05579 and PYU1_G013215 from *Py. ultimum* have N-terminal secretion signals. Secreted catalases have also been described in fungal pathogens [51]. While the two *Ph. infestans* genes were up-regulated 60-fold in early tubers compared to media (1012 vs. 8.7 aggregate CPM), PYU1_G013215 was down-regulated six-fold (178 vs. 32 CPM). All were expressed at low levels in late tubers.

The induction of *Ph. infestans* catalases during early infection is consistent with the observation that a catalase was induced early during infection of tobacco by *Ph. nicotianae* [52]. That the most infection-induced and most highly-expressed *Ph. infestans* catalase is secreted may suggest that its role is to eliminate H_2_O_2_ before it reaches the pathogen, or limit peroxide signaling within the plant [53]. While catalase CPM in early tuber was low in *Py. ultimum*, that species may be able to eliminate intracellular peroxides using other enzymes such as glutathione peroxidase.

#### NADPH oxidase

This generates the toxic compound superoxide. *Ph. infestans* and *Py. ultimum* are predicted to encode 12 and 21 NADPH oxidases, respectively, of which 10 and 19 were expressed at CPM ≥1.0 (Fig. 7b). As in animals, all of the predicted expressed proteins contain transmembrane domains and therefore this is not a secreted activity. Their aggregate CPM was higher in early tubers compared to media in both *Ph. infestans* and *Py. ultimum*, although more *Py. ultimum* genes were infection-induced. Total NADPH oxidase CPM was four times higher in *Py. ultimum* than *Ph. infestans* in early tuber.

These results are consistent with a model in which the pathogens produce superoxide to damage host enzymes, cell walls, or trigger programmed cell death [54]. Similar activities have been proposed for necrotrophic fungi such as *Sclerotinia sclerotiorum* [55]. This is consistent with the much higher CPM of the enzymes in *Py. ultimum.* With *Ph. infestans,* the effect of superoxide may be localized or its ability to trigger host cell death may be delayed by effectors during the biotrophic phase.

#### Glutathione S-transferase (GST)

This inactivates toxins by conjugating them to glutathione. *Ph. infestans* and *Py. ultimum* are predicted to encode 17 and 19 GSTs, respectively, of which 12 and 15 were expressed at CPM ≥1.0 (Fig. 7b). A similar fraction of genes were induced in both species during early infection compared to media, however the aggregate CPM was two times higher in *Py. ultimum* than *Ph. infestans.* This was because several *Ph. infestans* genes were down-regulated in early infection compared to media, and since one infection-induced *Py. ultimum* gene (PYU1_G000253, predicted to be cytoplasmic or peroxisomal) was expressed highly, accounting for 40% of total GST CPM. A reversal of this expression pattern occurred during late infection, when total GST CPM was two times higher in *Ph. infestans.*

These results are consistent with a role of GST in necrotrophic growth, i.e. all timepoints in *Py. ultimum* and the late timepoint in *Ph. infestans*. GST may protect pathogens by reducing endogenous or exogenous H_2_O_2_, other toxins of plant origin, and fatty acid peroxides. In *Alternaria brassicicola,* mutants deficient in GST showed higher sensitivity to plant isothiocyanates [56]. GST has also been implicated in protection against endogenous peroxides in both fungi [57] and plants [58].

### Nutrient uptake

Pathogens acquire most nutrients from the host using transporter proteins. The two pathogens allocate roughly the same proportion of their genes to these transporters, 2.6% for *Ph. infestans* and 3.0% for *Py. ultimum*. Overall, the number induced by at least three-fold in early tubers compared to media was slightly higher in *Py. ultimum* than *Ph. infestan*s (11.4% vs. 9.7%), while more were induced in late compared to early tubers by *Ph. infestans* (26.8% vs. 9.6%). Families showing the most changes are discussed in the next sections.

#### Amino acid/auxin permeases (AAAP)

**Fig. 8:**
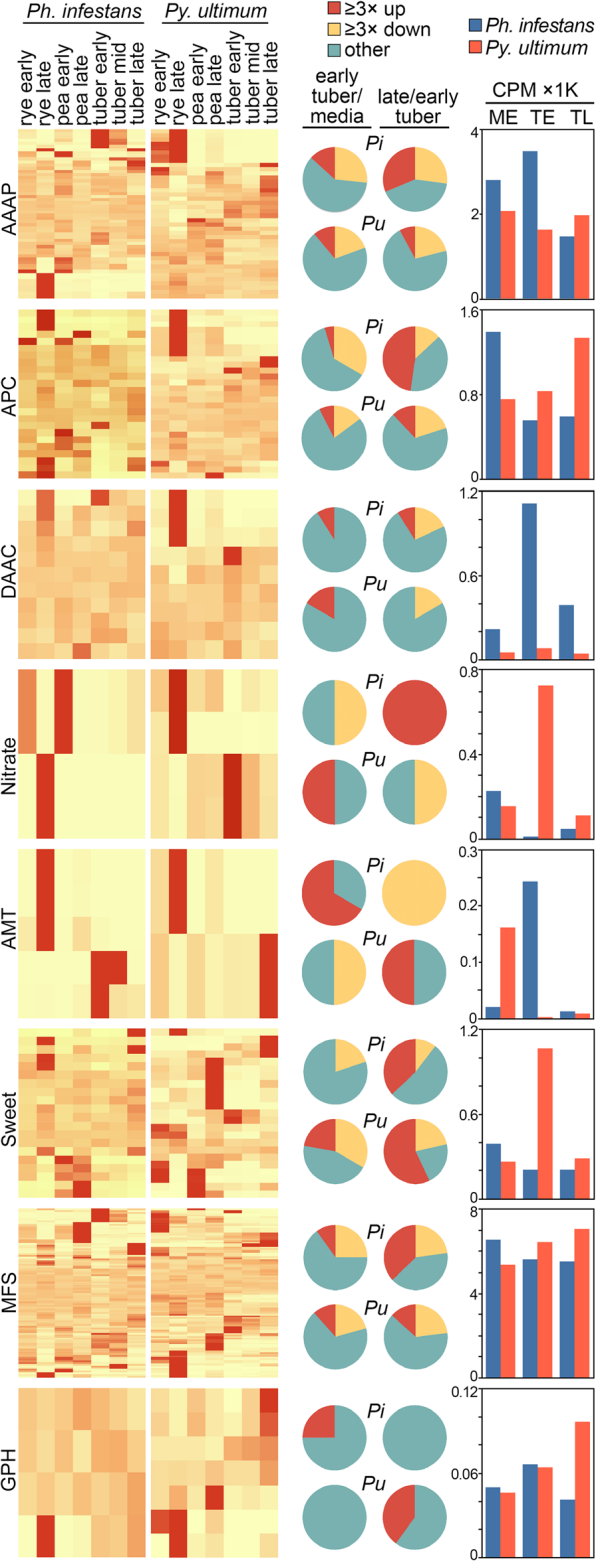
Expression of major gene families involved in nutrient transport. The format of the figure is the same as in Fig. 4, and functional categories are defined in the main text

*Ph. infestans* is predicted to encode 57 AAAPs compared to 49 in *Py. ultimum* (Fig. 8). Based on the CPM cut-off of 1.0, 54 *Ph. infestans* and 40 *Py. ultimum* AAAP genes were expressed in at least one of the conditions. While a similar number of genes in each species were up-regulated in early tubers compared to media (about 10%), the aggregate CPM of AAAPs in *Ph. infestans* was twice that of *Py. ultimum.* This was largely due to two highly expressed genes, PITG_20230 and PITG_12808. During the transition from early to late tubers, 28% and 8% of the *Ph. infestans* and *Py. ultimum* genes were up-regulated, respectively. Nevertheless, the aggregate CPM of AAAPs in late tuber was slightly higher in *Py. ultimum* than *Ph. infestans*, 1974 vs. 1470.

#### Amino acid/polyamine/organocation family (APC)

Both species encode similar numbers of these transporters, 27 for *Ph. infestans* and 30 for *Py. ultimum*, with 24 and 29 expressed at CPM ≥1 in at least one tissue, respectively (Fig. 8). Only one and two genes were up-regulated in *Ph. infestans* and *Py. ultimum* in early tubers compared to media, while ten and three were induced in late versus early tubers. Nevertheless, aggregate APC CPM levels in tubers were 44–250% higher in *Py. ultimum.* A large contributor was a single highly expressed gene, PYU1_G005219, which accounted for 56% of total transcripts in late tubers.

The APC and AAAP families are believed to represent the major plasma membrane transporters for amino acids in most organisms. Changes in expression during infection could be related to variation in the levels of extracellular amino acids caused by effector action in the case of *Ph. infestans*, or leakage from cells during *Py. ultimum* necrotrophy [59].

#### Dicarboxylate/amino acid:Cation symporter (DAACS)

Substrates of these also include amino acids, although these proteins often accumulate in both intracellular organelles and plasma membrane. *Ph. infestans* and *Py. ultimum* are predicted to encode 11 and 13 DAACS proteins, respectively, of which 11 and nine were expressed with CPM ≥1 in at least one tissue (Fig. 8). Only one gene was induced in early tubers versus media per species. While tissue-specific expression patterns in the two species were similar, this was not true for the aggregate CPM of DAACS which was five to 14 times higher across all tissues in *Ph. infestans.* The peak expression in early tuber was due to a single gene, PITG_09295, which accounted for 83% of total DAACS CPM.

#### Nitrate and ammonium transporters

These present alternative routes for acquiring nitrogen. *Ph. infestans* encodes four predicted nitrate transporters, of which two had a CPM ≥1. The heatmaps in Fig. 8 indicate that these are expressed more in late than early tubers. However, based on CPM these represent very low transcript levels, two orders of magnitude lower than what occurs in potato and tomato leaves [60]. Nitrate levels are generally high in leaves and low in tubers.

The expression of nitrate transporters was very different in *Py. ultimum.* Its four predicted transporters were all expressed, with aggregate nitrate transporter CPM 100-times that of *Ph. infestans* in early tuber. This was largely due to PYU1_G001247, which at 695 CPM accounted for 95% of total nitrate transporter CPM. In media and late tubers, the aggregate CPM of *Ph. infestans* and *Py. ultimum* were similar at 46 and 111 CPM, respectively.

The expression of ammonium transporters (AMTs) was also strikingly different between the species. However, in this case the aggregate AMT CPM in *Ph. infestans* was 100-times higher than *Py. ultimum* in early tubers. Of five predicted AMTs in *Ph. infestans,* two were expressed only in tubers and three in artificial media. Similarly, one of the two predicted *Py. ultimum* AMTs was largely tuber-specific and the other media-specific.

The opposing patterns of expression of these transporters may reflect a greater reliance of *Py. ultimum* on nitrate, and of *Ph. infestans* on ammonium. In soil, nitrate is more abundant and thus more useful to *Py. ultimum* during its saprophytic stage. In contrast, ammonium occurs throughout plant tissues and should be more useful to the more host-dependent species, *Ph. infestans* [60]; while *Ph. infestans* can use ammonium as the sole nitrogen source, it is not as favorable as amino acids [61]. Alternatively, the main role of the transporters may be for efflux or to alkalinize and weaken host tissues, not to acquire nitrogen for new biomass [62].

#### SWEET transporters

*Ph. infestans* and *Py. ultimum* are predicted to encode 32 and 28 SWEET transporters, of which 28 and 20 were expressed at CPM ≥1, respectively (Fig. 8). In plants, these participate in the uptake and efflux of mono- and disaccharides [63]. Presumably these are used for sugar uptake by the pathogens.

In *Ph. infestans,* the aggregate CPM of SWEETs was similar in all tissues, although a few genes were induced during late infection. In contrast, the aggregate CPM of SWEETs in *Py. ultimum* was three-fold higher in early infection than the other stages. Despite the fairly large size of the SWEET family in *Py. ultimum,* 95% of total SWEET CPM in early tubers came from a single gene, PYU1_G003519. This belongs to a cluster of 10 genes, of which eight were not expressed in any tissue. *Ph. infestans* SWEET genes also occurred in clusters, with a majority also lacking expression in the tissues examined.

#### MFS transporters

This superfamily of secondary transporters was originally thought to only participate in sugar uptake, but was shown later to carry a broader spectrum of substrates [48]. MFSs represent nearly a quarter of the nutrient transporters encoded by *Ph. infestans* and *Py. ultimum*, with 112 and 118 genes in the two species, respectively. Of these, 90 and 88% were expressed at CPM ≥1, respectively (Fig. 8).

A similar number of *Ph. infestans* and *Py. ultimum* MFS genes were induced during early infection compared to media, although the aggregate MFS CPM was fairly constant in all tissues and similar between species. Three genes in each species were expressed specifically in tubers compared to media, with half predicted to be hexose transporters (PYU1_G009043 and G009044, PITG_12998).

#### Glycoside-pentoside-hexuronide family (GPH)

This family is believed to be distantly related to the MFS group. In symport with a monovalent cation, they transport a range of carbohydrates that are mostly but not exclusively glycosides [64]. *Ph. infestans* and *Py. ultimum* are predicted to have eight and nine GPH genes, of which four and seven were expressed at CPM ≥1 (Fig. 8). Apart from one *Ph*. *infestans* gene induced in early tuber (PITG_06336) and two *Py. ultimum* genes induced in late tuber (PYU1_G002035, G002036), most were expressed constitutively.

The aggregate CPM values of GPH proteins were 10 to 100 times lower than MFS and SWEET transporters. It thus appears that the MFS and SWEET families bear the primary responsibility for sugar uptake in both pathogens. For *Ph. infestans,* this may entail competition with plant apoplastic transporters, or help reduce leakage of sugars from the pathogen to the apoplast, which may trigger plant defenses [65]. Some of the proteins may also serve as sugar sensors and not transporters*,* as observed in *Colletotrichum* [65].

### Global analysis of variation in ortholog expression levels

Above, we described interspecific differences in the level and stage-specific patterns of transcription of genes in functional categories relevant to plant colonization. Only in select cases did family expansions explain the variation in mRNA levels. When all *Ph. infestans* and *Py. ultimum* genes were examined, 21% of families were found to vary in size by two-fold or more; 903 families had >2-fold more genes in *Ph. infestans* and 553 had >2-fold more in *Py. ultimum* (Additional file 4: Figure S2). However, as shown in Additional file 5: Figure S3, the correlation between family size and expression level was weak in *Py. ultimum* (e.g. *R* = 0.42 in rye media) and nonexistent in *Ph. infestans* (*R* = 0.03). This is because many family members were often expressed at low levels, as illustrated for polyphenol oxidases in Fig. 3 and NPP1 proteins in Additional file 3: Figure S1.

To better address the extent of transcriptional remodeling between *Ph. infestans* and *Py. ultimum,* we compared orthologs genome-wide. To minimize error, genes were defined as orthologs only if consistent assignments of orthology were obtained using the OrthoMCL and InParanoid pipelines, and the reciprocal best hit approach [66–68]. To avoid assigning Illumina reads to the wrong gene, genes were excluded if they contained regions >98% identical to another family member. This resulted in the identification of 5559 “high-confidence” ortholog pairs, which included 3945 single-copy genes and 1614 belonging to families.

**Fig. 9:**
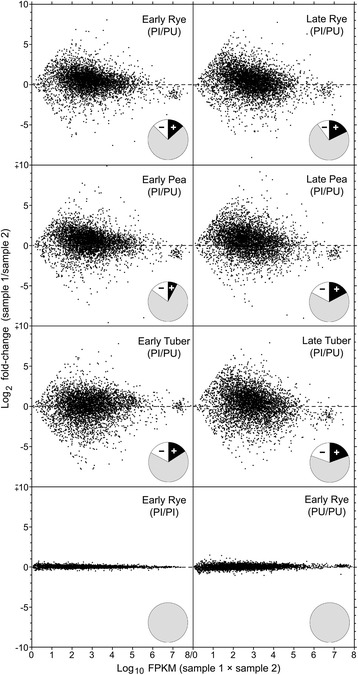
MA plots of ortholog pairs. The y-axis indicates the log_2_ ratio of the FPKM values of the two samples being compared (M) while the x-axis shows the log_10_ product of their FPKM values (A). The top six panels compare *Ph. infestans* (Pi) and *Py. ultimum* (Pu) in early and late rye, pea, and tuber. The bottom two panels compare two replicates of *Ph. infestans* and *Py. ultimum* in early rye. The pie charts indicate the fraction of genes that are up- (+) or down-regulated (−) in *Ph. infestans* compared to *Py. ultimum* based on a fold-change threshold of 3.0. Only genes with FPKM ≥ 1 in at least one species are shown

Frequent divergence in expression level, measured as FPKM (fragments per kilobase per million mapped reads), was observed between *Ph. infestans* and *Py. ultimum* orthologs in all tissues (Fig. 9). For example, more than three-fold divergence was observed in early tuber between 1732 orthologs, or 34% of genes expressed at that stage. Slightly less divergence occurred in artificial media, and slightly more in late samples, the latter due possibly to the effects of sporulation. The differences between species were not attributable to experimental variation, because only minor changes were observed between biological replicates. The degree of divergence of single-copy genes and those belonging to families was equivalent. Despite the significant divergence of individual genes, the expression levels of most genes were fairly similar, with Pearson’s correlation coefficient calculated at 0.88, 0.87, 0.91, 0.71 and 0.80 in early rye, early pea, early tuber, late rye, and late tuber, respectively. There was no significant correlation in randomly shuffled data (*R* = 0.01 to 0.05).

### Global analysis of variation in stage-specific patterns of orthologs

Besides divergence in mRNA level, divergence was observed in tissue-specific patterns of expression. As illustrated in Additional file 6: Figure S4a for a comparison between early tuber and media, 833 ortholog pairs were differentially expressed in only one species and 52 in opposite directions, i.e. up-regulated in one species and down-regulated in the other. The definition of “differentially expressed” used here is a three-fold change. One interesting example involved secreted catalases. While *Ph. infestans* gene PITG_07143 was induced 164-fold in early tuber compared to media, its *Py. ultimum* ortholog, PYU1_G013215, was down-regulated six-fold. Another example involved acid phosphatases. While *Py. ultimum* gene PYU1_G000880 was induced 16-fold in early tuber, its *Ph. infestans* ortholog, PITG_17872, was down-regulated eight-fold. The other divergently expressed genes are listed in Additional file 7: Table S3.

A comparison of ortholog pairs in late and early tubers showed more variation, as 2012 were up or down-regulated only in one species, and 69 were up-regulated in one species but down-regulated in the other (Additional file 6: Figure S4b; Additional file 8: Table S4). Orthologs that were up-regulated in late tubers only in *Py. ultimum* had diverse roles that included amino acid catabolism, gluconeogenesis, and dehydrogenase reactions. Genes that were up-regulated only in *Ph. infestans* often encoded flagella-associated proteins. Genes that were up-regulated in one species but down-regulated in the other included many involved in sulfate metabolism, including sulfate transporters and sulfite reductase.

Some of these genes, such as the catalases, fall into categories shown in earlier sections to vary between the two oomycetes. However, this analysis draws a distinct conclusion. Patterns of gene expression have changed not only due to gene gain or loss, or family expansions, but due to the transcriptional remodeling of orthologs. While this may be due to changes in promoters or their cognate transcription factors, it is possible that each species is responding to environmental changes attributable to their biotrophic and necrotrophic lifestyles, such as levels of toxic plant compounds.

### Conservation of expression and sequence are positively correlated

**Fig. 10:**
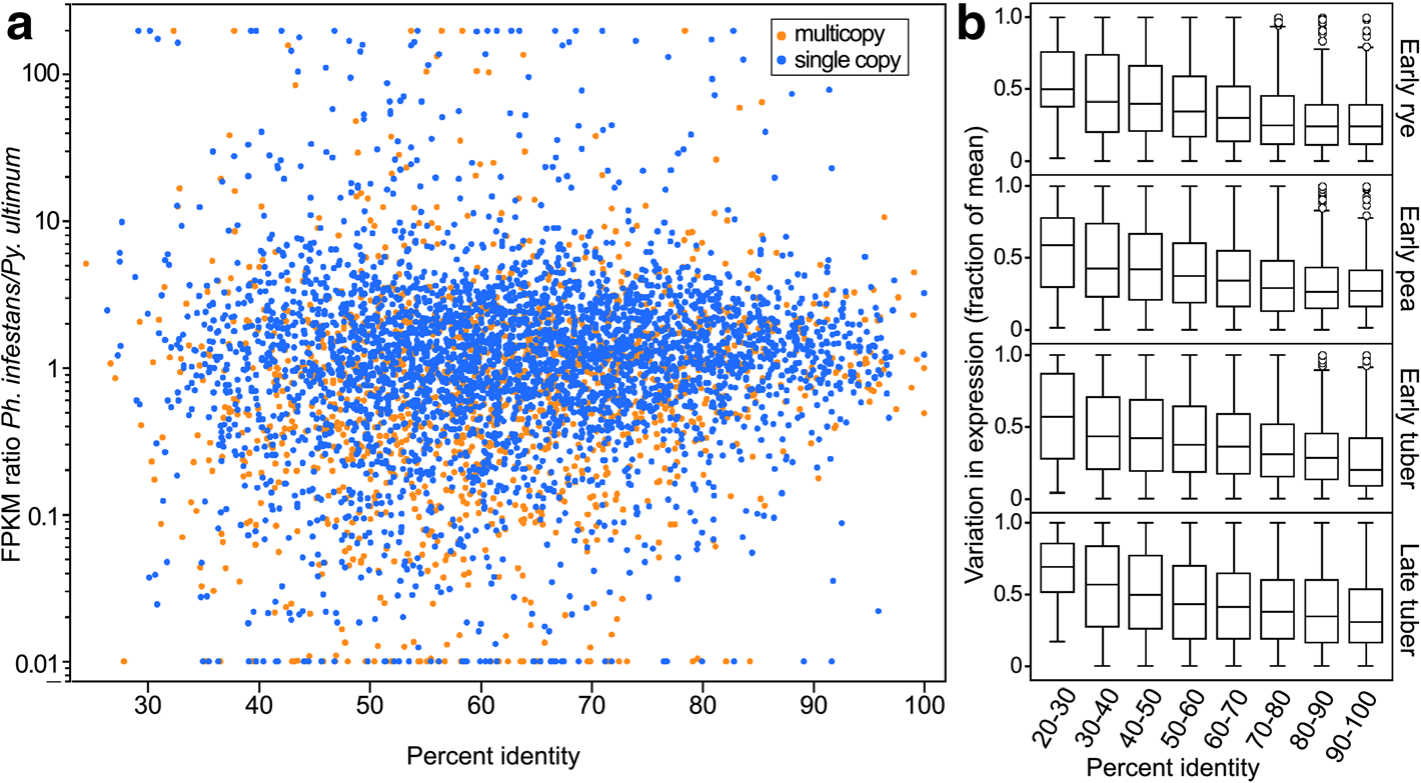
Relationship between amino acid similarity and expression conservation. **a** Plot of FPKM ratio of orthologs (*Ph. infestans* divided by *Py. ultimum*) in early tuber versus amino acid identity. Orange and blue symbols represent members of multigene families and single-copy genes, respectively. **b** Box-plots indicating variation in expression level in early rye (based on 5273 genes with FPKM ≥ 1), early pea (5211 genes), early tuber (5123 genes), and late tuber (5461 genes) as a function of amino acid identity. Similar trends were seen in late rye and pea. Variation is defined as the difference between the FPKM of orthologs divided by their summed FPKM values. Ratios above 200 or below 0.01 are graphed as 200 and 0.01, respectively

Most studies have found that the divergence of expression between orthologs is largely independent of protein similarity [69–71], although studies of mouse and human have yielded contradictory results [72, 73]. To test this in oomycetes, a genome-wide analysis was performed by graphing the FPKM ratio of *Ph. infestans* and *Py. ultimum* orthologs versus amino acid identity (Fig. 10). This showed that variation in expression level increased with reduced amino acid identity in all growth conditions, for both single-copy genes and members of families. Our strict rules for assigning orthologs may have, however, eliminated many genes having more divergent expression.

### Variation in orthologs based on functional classification

**Fig. 11:**
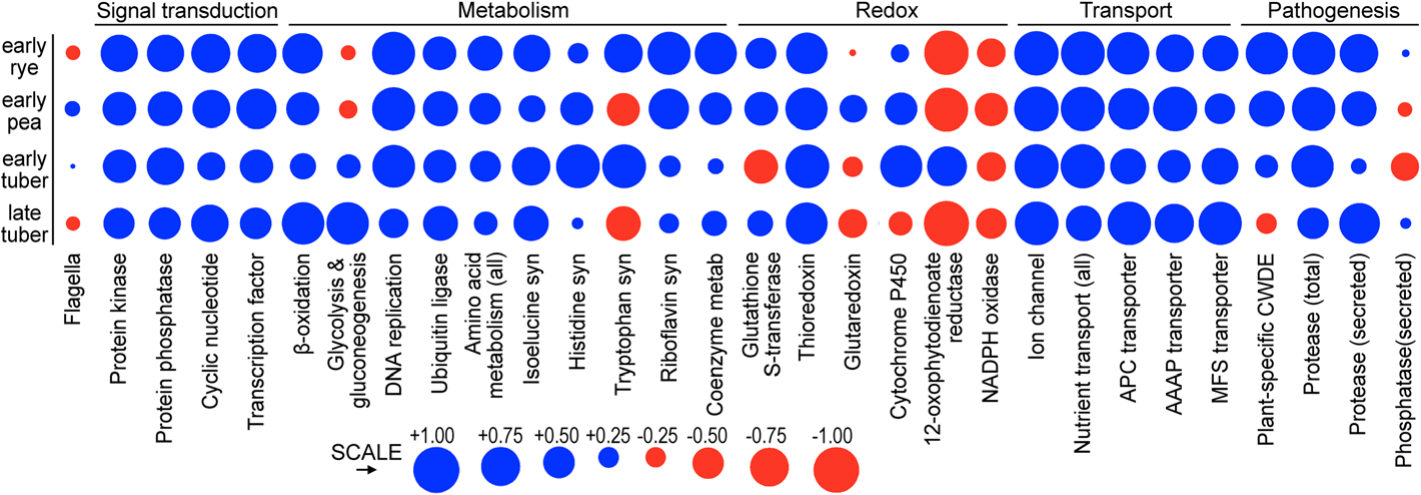
Correlation between FPKM of orthologs in selected functional groups. Ortholog pairs were classified into functional groups and filtered to include only those with FPKM ≥ 1 in both species. Pearson’s correlation coefficients are graphed as positive (blue) or negative (red) in early rye and pea, and early and late tuber. The area of each circle is proportional to the degree of correlation

We also tested for correlations in the expression of orthologs categorized by protein function (Fig. 11). The results indicated that orthologs associated with most core cell functions had well-correlated expression values in all conditions. These included genes with roles in DNA replication, ion channels, signal transduction, and amino acid metabolism with the exception of tryptophan and histidine synthesis. Negative correlations were observed for NADPH oxidase, secreted phosphatases, and others. In categories such as CWDEs, secreted proteases (but not all proteases), cytochrome P450s, and glutaredoxin, the divergence was most pronounced during tuber infection. As in prior analyses, the differences may be due to transcriptional remodeling of orthologs or specific responses of each species to environmental changes attributable to their lifestyles.

An independent method for measuring mRNA levels confirmed that the negative correlations in expression between the species were not artifacts of the RNA-seq technique. This involved using reverse transcription quantitative polymerase chain reaction (RT-qPCR) against six representative orthologous pairs of genes that RNA-seq indicated were expressed discordantly (Additional file 9: Figure S5). In each case, the conclusions from RNA-seq were upheld by RT-qPCR. For example, one cause of the negative correlation for tryptophan synthesis in late tubers were genes PYU1_G005607 and PITG_01711. These are single-copy, syntenic genes encoding anthranilate synthase. In RNA-seq of late tubers, the *Py. ultimum* gene was expressed seven times higher than its *Ph. infestans* ortholog, with FPKM values equaling 1382 and 205, respectively. The same trend was observed in RT-qPCR, which measured 98 and 14 mRNA copies per pg of cDNA in *Ph. infestans* and *Py. ultimum,* respectively, after correcting for the fraction of total RNA from each pathogen in the two samples.

### Family-wide analysis of transcriptional remodeling

**Fig. 12:**
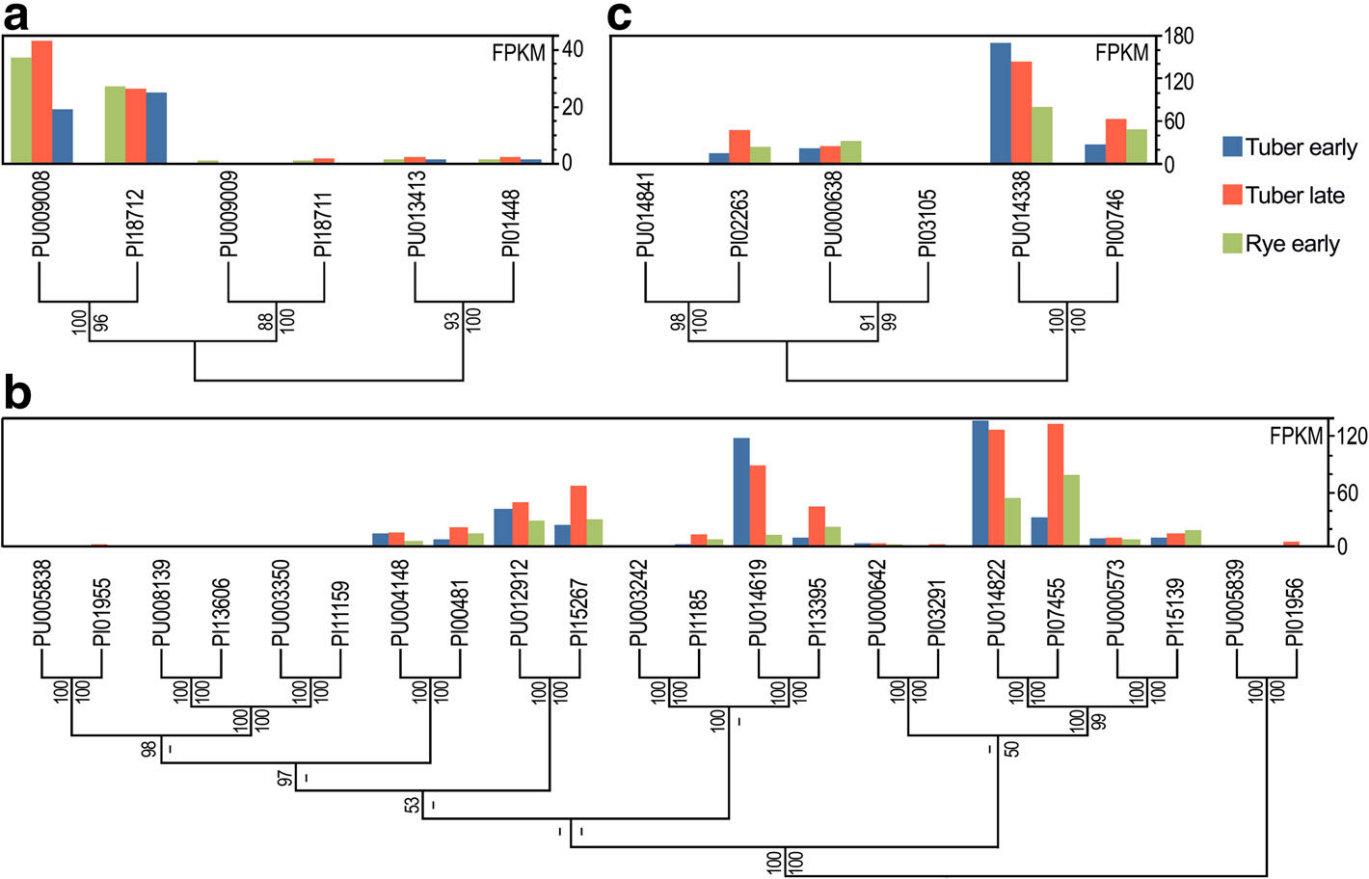
Examples of conserved and discordant expression patterns within orthologous families. Illustrated are three families of orthologs encoding **a** a regulatory subunit of calcineurin; **b** P-ATPases; and **c** a metal ion transporter. Indicated on the left side of each panel are cladogram trees with bootstrap values from PhyML (top) and neighbor-joining. The *Ph. infestans* and *Py. ultimum* gene numbers have PI and PU prefixes, respectively. The bar charts above the trees indicate FPKM of each gene in early tuber, late tuber, and early rye

The frequency of shifts in the expression of orthologs was addressed by studying 55 OrthoMCL groups in which both species had equal numbers of paralogs and phylogenetic analysis supported the ortholog assignment. In 76% of the families, expression levels and patterns were well-conserved within each ortholog pair. An example is in Fig. 12a, which portrays a small family of calcineurin subunit genes. One ortholog pair was expressed at relatively high levels in all tissues (PITG_18712, PYU1_G009008), while the other two pairs shared low expression. Figure 12b illustrates a second, larger family encoding P-ATPases, where expression levels and patterns within each ortholog pair are also generally well-conserved.

In contrast to these examples, expression patterns or levels were conserved poorly in 24% of gene families. An example is in Fig. 12c, which shows a family of metal ion transporters. While one ortholog pair (PITG_00746, PYU1_G0144338) shows high expression in each tissue, expression of the other orthologs has been shuffled. In one ortholog pair, PITG_02263 shows moderate expression while zero expression was seen for PYU1_G014841. In a second ortholog pair, PYU1_G000638 and PITG_03105 show moderate and zero expression, respectively. The ortholog assignments in this family are supported by synteny. The transcriptional network that regulates the family thus appears to have become rewired during evolution.

### Genome-wide analysis of pseudogenization

#### Pseudogenes in families shared by Ph. infestans and Py. ultimum

Many genes in both oomycetes lacked a CPM >1.0 in any of the six growth conditions, and were thus possible nontranscribed pseudogenes. This threshold was selected to avoid counting spurious transcripts, and corresponded to about 20 reads per average gene in our study. In mammals, a CPM of 1.0 equals about 1 transcript per nucleus [74]. Excluding RXLR and CRN genes, 25 and 15% of *Ph. infestans* and *Py. ultimum* genes in the functional groups described earlier in this paper were unexpressed. The difference between species was significant at *p* = 10^−6^ by Fisher’s exact test. Possibly contributing to the greater fraction of unexpressed genes in *Ph. infestans* was its increased propensity towards expansion of gene families (Additional file 4: Figure S2). This is commonly associated with pseudogenization or regulatory subfunctionalization, in which genes acquire new expression patterns [2].

As an initial test of whether genes with low CPM had undergone pseudogenization, regulatory subfunctionalization, or were simply expressed under growth conditions not represented so far in this study, we examined the *NPP1* family of *Ph. infestans.* Earlier we described how the family had expanded from seven genes in *Py. ultimum,* all expressed, to 55 in *Ph. infestans,* of which 33 had CPM <1. By examining RNA-seq data from five additional developmental stages (purified asexual sporangia, sporangia chilled to differentiate zoospores, swimming zoospores, germinating cysts making appressoria, sexual spores [24]) and a fungicide treatment (0.5 μg/ml metalaxyl), 12 additional genes were deemed to be expressed. The remaining 20 *NPP1* genes are thus candidate nontranscribed pseudogenes*.* All but one had matching sequences in an assembly of genomic reads from strain 1306.

**Fig. 13:**
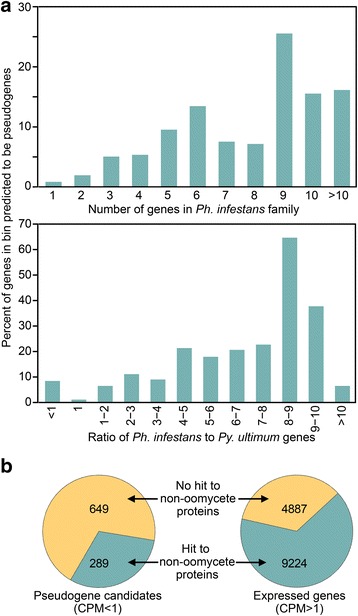
Pseudogenization in *Ph. infestans.* **a** relationship between occurrence of a candidate pseudogene and either the size of the gene family (top) or the ratio of paralog numbers in *Ph. infestans* and *Py. ultimum* (bottom). **b** comparison of the fraction of pseudogenes and expressed genes having hits in GenBank against non-oomycete proteins

Next, we performed a genome-wide assessment of whether pseudogenization was associated with the size of a gene family*.* This focused on genes that contained orthologs in both species, a strategy that would exclude false gene models. By checking for expression in all 12 growth conditions or life stages, we identified 938 candidate pseudogenes. A gene was more likely to be a pseudogene if it belonged to a large family or one that was expanded in *Ph. infestans* compared to *Py. ultimum* (Fig. 13a). A gene was also more likely to be a pseudogene if it was oomycete-specific (Fig. 13b). The difference was significant at *p* = 10^−96^ by Fisher’s exact test. Of the 938 candidates defined by mapping RNA-seq reads to the T30–4 reference genome, nine lacked matching sequences in the genome assembly of strain 1306, and 32 showed enough divergence to impede the mapping of the reads. Therefore, the number of candidate nontranscribed pseudogenes in families shared with *Py. ultimum* is 897.

We also checked for evidence of regulatory subfunctionalization in families common to *Ph. infestans* and *Py. ultimum* by checking for genes that were expressed only during asexual sporulation, zoosporogenesis, or spore germination in *Ph. infestans*. Fitting this criteria were 1574 genes, although it is difficult to claim definitively that these are not sporulation-induced in *Py. ultimum* since whether the latter makes spores is controversial [14, 15]. Genes in *Ph. infestans* were more likely to be associated with spores if they had more than two genes per family (*p* = 10^−47^ by Fisher’s exact test), or were in a family that was expanded in *Ph. infestans* relative to *Py. ultimum* (*p* = 10^−69^). This is consistent with the occurrence of subfunctionalization and neofunctionalization in other taxa [2, 27].

#### Other pseudogenes

Also lacking expression in the 12 growth conditions and life stages were 738 *Ph. infestans* genes that lacked orthologs in *Py. ultimum.* Of these, 89 were predicted to encode RXLR proteins and 243 had matches to proteins from other *Phytophthora* spp. in BlastP with *E* < 10^−20^. The remainder, 340, may be false gene models. Eliminating those, there are 1295 candidate pseudogenes in *Ph. infestans* strain 1306. These are listed in Additional file 2: Table S5.

By combining *Py. ultimum* RNA-seq data from this study with samples from a prior report that focused on heat and chemical stress [17], we identified 1565 gene models that lacked CPM ≥1.0. By filtering for genes containing matches in other Pythium species, a tentative conclusion is that 1203 are candidate pseudogenes and the rest may be false gene models.

#### Genomic architecture and pseudogenes

Oomycete genomes contain gene-dense and gene-sparse regions. Since gene evolution is reportedly accelerated in the latter [16], the genomic distribution of the pseudogene candidates of *Ph. infestans* was analyzed*.* The average physical distance between pseudogenes and neighboring genes was 18% more than for expressed genes. Pseudogenization of sequences in the gene-sparse regions was enhanced, but so was the generation of pseudogenes from tandemly repeated families in gene-dense regions. Twenty percent of pseudogene candidates were adjacent to an expressed member of the same family, and 30% were within four genes of an expressed member of the family.

### Species-specific genes

A sign of genome evolution other than divergence of orthologs or gene family expansions are species-specific genes, which may originate through gene loss or gain, including horizontal gene transfer. The RXLR and Crinkler families are one well-studied example in oomycetes, which are abundant in *Phytophthora* but not *Pythium* genomes [17]. Little data are available about other species-specific genes. We therefore identified and studied the expression patterns of genes that lacked relatives in the other species, using a maximum *E*-value threshold of 10^−25^ in BlastP and a minimum protein size of 50 amino acids.

#### Genes specific to Ph. infestans

These numbered 2801, excluding RXLRs and Crinklers (Additional file 2: Table S6). Expressed based on the CPM threshold of 1.0 were only 1209 genes, which suggests that many may be false gene models. Of the expressed genes, 136 encoded secreted proteins. Up-regulated in early and late tuber stages, respectively, were 9% and 38% of genes encoding nonsecreted proteins, and 35% and 30% of those encoding secreted proteins.

Most of the predicted secreted proteins represent categories described previously including protease and glucanase inhibitors, small cysteine-rich proteins, cutinase, and berberine bridge enzymes [31, 41, 75]. Contrary to the findings of Ospina-Giraldo et al. who examined leaf infections [76], we found that cutinases were induced only in late tubers. The discrepancy could be due to the absence of a cuticle in tubers, or the possibility of suberization in our tuber slices [77]. Like some fungal cutinases, the *Ph. infestans* enzymes might be able to degrade both cutin and suberin [78].

Several intracellular metabolic enzymes were also specific to *Ph. infestans,* including gluconolactonase, sorbitol dehydrogenase, and pyruvate phosphate dikinase. All were highly expressed *in planta*. The latter, which acts at the final step of glycolysis, has a lower free energy than the alternate pyruvate kinase enzyme, and may thus facilitate gluconeogenesis by being more reversible [79]. The sorbitol dehydrogenases might contribute to osmotic balance or quench reactive oxygen. The glucolactonase might contribute indirectly to the saccharification of cellulose to glucose by eliminating a known inhibitor of β-glucosidase [80].

Prior studies identified an early infection-specific gene, *Hmp1,* which encodes a protein of unknown function bearing a signal peptide and transmembrane domain targeted to haustoria [81]. A search of the *Ph. infestans*-specific genes for proteins with the same expression pattern and structural features identified three: PITG_06212, PITG_19140, and PITG_16084. Whether these might also be haustorial remains to be determined, although each lacked sequence similarity to Hmp1.

#### Genes specific to Py. ultimum

Identified were 452 genes, of which 322 were transcribed at CPM ≥1 (Additional file 2: Table S6). Sixty-one of the total are predicted to encode secreted proteins, of which 43 were expressed. Up-regulated in early and late tuber stages were 12% and 8.4% of the 322 genes, and 30% and 14% of expressed secreted genes, respectively.

One gene, PYU1_G000106, encodes a protein not described previously in an oomycete (Additional file 10: Figure S6a). The 588-amino acid protein exhibits a strong match (*E* = 10^−42^) to the 394-amino acid membrane-attack complex/perforin (MACPF) domain. In plants and animals, MACPF proteins form pores in cell membranes, leading to programmed cell death in development or defense against pathogens [82, 83]. In apicomplexans, the proteins are secreted virulence factors [84]. The *Py. ultimum* gene contains a predicted signal peptide (SignalP score of 0.94), consistent with its affinity in phylogenetic analyses to apicomplexan proteins. The *Py. ultimum* gene is barely expressed in media (averaging 0.6 CPM), but rises to 1.9 in early tuber and 10 CPM in late tuber (Additional file 10: Figure S6b). We find MACPF orthologs in most *Pythium* spp., but no other oomycete (including both saprolegnians and peronosporaleans) or other stramenopile. The *Pythium* proteins showed the highest amino acid similarity to apicomplexan MACPF, although the *Pythium* and apicomplexan proteins did not form a well-supported clade in phylogenetic studies (Additional file 10: Figure S6c).

One metabolic enzyme specific to *Py. ultimum* was the starch-degrading protein glucoamylase. Full utilization of this plant (but not oomycete) storage molecule requires α-amylase, which cleaves α-1,4 bonds in starch, and glucoamylase, which breaks its α-1,6 linkages. *Ph. infestans* and *Py. ultimum* encode one and three α-amylases, respectively, but only *Py. ultimum* encodes glucoamylase. This explains why starch is an inferior carbon source for *Phytophthora* [85], but a good substrate for *Pythium* [40]. Glucoamylase is encoded by a cluster of four genes in *Py. ultimum*, all expressed and encoding secreted proteins. Their aggregate CPM averaged 124 CPM in media, and rose from 65 in early tuber to 331 in late tuber. The α-amylases of *Py. ultimum* showed a much greater increase during infection, having 1.5 CPM in media, 8.0 in early tuber, and 99 in late tuber. In contrast, the *Ph. infestans* α-amylase was 0.8 CPM in media, 0.2 in early tubers, and 4.8 CPM in late tubers. Thus, it seems that the superior ability of *Py. ultimum* to use starch is due to its gain of glucoamylases (or loss from *Ph. infestans*), three-fold expansion of its α-amylase family relative to *Ph. infestans,* and transcriptional remodeling that increased the CPM of each of its α-amylase genes by about five-fold compared to single *Ph. infestans* gene.

Another feature of metabolism unique to *Py. ultimum* is the presence of N-acetylglucosamine-6-phosphate deacetylase (PYU1_G006885) and glucosamine 6-phosphate isomerase (PYU1_G006886). These convert chitin into glucose-6-phosphate, which can enter glycolysis. Both are expressed in media and tubers, with the latter doubling in CPM during late infection. Interestingly, the two genes are neighbors in *Py. ultimum* and five other members of the genus. They are not linked in ortholog-containing eukaryotes (fungi and animals), but are often adjacent in bacteria [86]. There is however poor support for horizontal gene transfer from bacteria. Orthologs are found in *Blastocystis*, which like oomycetes belongs to the stramenopile kingdom. However, the genes are absent in other stramenopiles including diatoms and saprolegnian oomycetes, and the *Blastocystis* genes do not form a strong cluster with the *Pythium* genes in phylogenetic analysis.

## Discussion

This study had the dual goals of revealing differences between biotrophic and necrotrophic growth, and of how transcriptomes evolved in oomycetes having these divergent lifestyles. Differences between *Ph. infestans* and *Py. ultimum* included species-specific genes, variation in ortholog expression, and changes in gene family size. Interestingly, expansions within families were often not reflected in aggregate transcript levels, which could be due to divergence in promoter activity, mRNA stability, or epigenetic repression. Changes within promoters pseudogenized many genes, as reported within gene families in non-oomycetes [87]. Variation in promoters may also have shifted activity to life stages not shared between *Ph. infestans* and *Py. ultimum* such as haustoria or sporulation*.* This phenomenon, known as regulatory subfunctionalization, can occur through alterations in promoter sequence, the binding specificity of cognate transcription factors, or the regulation of those factors [2]. Lévesque et al. [17] noted that gene orders in *Py. ultimum* and *Ph. infestans* were often inverted*,* which could affect their 5′ regulatory regions.

Several trends relevant to the issue of biotrophic versus necrotrophic growth were seen in our data. One is that more genes were expressed constitutively in *Py. ultimum* than *Ph. infestans.* This was a feature of genes having orthologs in the two species such as catalases, pectate lyases, and many amino acid transporters, as well as species-specific proteins such as RXLR effectors. One explanation is that *Ph. infestans* deploys its genes in stages in order to produce haustoria, secrete effectors, and acquire nutrients at specific phases of infection. *Ph. infestans* may also need to balance the expression of effectors that suppress plant defenses with proteins that reduce plant cell integrity. In contrast, as a soil-borne necrotroph *Py. ultimum* goes “full speed ahead” with its pathogenic program. This may help *Py. ultimum* compete with other microbes for nutrients.

While variation in the timing and level of ortholog expression may underlie many of the distinctions between biotrophy and necrotrophy, some of these differences could reflect secondary effects. For example, the higher expression of ABC and SWEET transporters in *Py. ultimum* may be responses to toxins or nutrients released from plant cells during necrotrophy, respectively. Similarly, some infection-induced *Ph. infestans* genes such as RXLR effectors or catalases may be responding to plant defenses unique to its interaction with tubers. Analyzing plant defense responses was beyond the scope of this analysis, but it is likely that there are substantial differences in the responses of tubers to the two pathogens, especially considering the rapidity at which *Py. ultimum* killed host cells. It should be noted that whether *Ph. infestans* is programmed to kill hosts cells late in infection is controversial; hyphae within necrosing host lesions are usually vacuolated, and sometimes the first macroscopic sign of infection is sporulation [88]. Nevertheless, late in infection *Ph. infestans* up-regulates the necrosis-inducing protein *NPP1*, which in contrast is expressed at its highest levels by *Py. ultimum* in early tubers.

The *NPP1* family also provided evidence of selection on gene families, influenced by the biotrophic or necrotrophic lifestyles. In *Py. ultimum,* six of its seven predicted *NPP1* genes were expressed and all contained the amino acid residues predicted to be required to cause host necrosis [36]. In contrast, only 22 of the 55 genes in *Ph. infestans* were expressed and most lacked the requisite amino acid residues. Thus, the expansion of the *NPP1* family in *Ph. infestans* was followed by pseudogenization or diversification of most of its members.

The *NPP1* family is just one of several that were differentially expanded between the two species. Another example were hemicellulases. These are encoded by 12 genes in *Ph. infestans,* which were expressed mostly during early infection, but by only two genes in *Py. ultimum,* which were expressed primarily during late infection. The timing of expression in *Ph. infestans* is consistent with the suggestion by Blackman et al. [41] that hemicellulose degradation by *Ph. parasitica* on lupin starts during early infection. In contrast to hemicellulases, the cellulase gene family was expanded only slightly in *Ph. infestans,* which encoded 13 proteins compared to nine for *Py. ultimum* (excluding those with GPI anchors)*.* However, the *Ph. infestans* genes were transcribed at much higher levels than the *Py. ultimum* genes. This illustrates the point that comparative studies need to consider both gene copy number and expression level.

The results with cellulase and hemicellulase suggest that large-scale wall saccharification is not a major feature of pathogenesis by *Py. ultimum* prior to late infection [40], and that that cellulose (the primary component of the plant wall) may be a significant carbon source only when other substrates such as simple sugars become limiting. Nevertheless, the higher expression of cellulases by *Ph. infestans* in its biotrophic early tuber stage compared to *Py. ultimum* was surprising, since *Phytophthora* spp. are thought to employ stealthy infection strategies that limit host damage [38]. The early-induced *Ph. infestans* cellulases may be used to make focused digests in plant walls to allow haustorial development or increase its susceptibility to later digestion. The staged expression of cellulases was also reported for *Ph. parasitica* [41].

Species-specific genes also appeared to contribute to the lifestyles of both species. The presence of glucoamylases only in *Py. ultimum,* for example, may reflect its preference for degrading simple carbohydrates. The presence of several chitin-degrading enzymes in *Py. ultimum* is consistent with its ability to grow as a saprophyte as well as a plant pathogen. While chitin is not made by potato and is but a minor component (<1%) of *Pythium* cell walls, it comprises a major fraction of soil organic matter [89, 90]. The most prominent family of *Ph. infestans*-specific genes, RXLR effectors, presumably help suppress the host necrosis that is a feature of *Py. ultimum* infections [38]. Conversely, proteins specific to *Py. ultimum* such as MACPF/perforin may accelerate host death in order to liberate nutrients during necrotrophic growth. The loss of this ancient cell-lysing protein from biotrophic or hemibiotrophic peronosporaleans such as *Ph. infestans* is consistent with the theory that biotrophy is more evolutionarily advanced than necrotrophy [1].

It should be noted that our analyses have considered biotrophy and necrotrophy only from the pathogen’s perspective. Since the lifestyle of a pathogen is integrated with its host, a better understanding would result from incorporating into the research gene expression patterns by the infected tubers as well as levels of metabolites. The latter would include both nutrients available to the pathogen and host defense compounds, such as reactive oxygen and phytolalexins. Particularly in a species such as *Ph. infestans* in which the disease cycle involves dynamic changes in gene expression, this would indicate which changes are hard-wired into the pathogen’s developmental program and which occur in response to metabolic transformations in the host. Integrated dual RNA-seq and metabolite fingerprinting have proved useful for studying fungal-plant interactions [91].

## Conclusions

The oomycetes *Ph. infestans* and *Py. ultimum* have undergone major shifts in genome composition and expression during evolution. Although the total number of their predicted genes differ by only about 17% [17, 18], individual families have undergone significant expansions or contractions plus transcriptional changes. Much of the latter appears to be linked to sub- or neo-functionalization or pseudogenization. The extensive families combined with divergent patterns and/or levels of expression within each family necessitated consideration of both the expression of individual genes and the aggregate expression of each family in our analyses. A conclusion from the research is that biotrophy and necrotrophy seem determined by species-specific genes and the varied expression of shared pathogenicity factors. The latter may be useful targets for crop protection.

## Methods

### Growth of pathogens

*Py. ultimum* var. *ultimum* was isolated from a potato farm in San Jacinto, CA and maintained at 21 °C in the dark on half-strength V8 medium containing 1.5% agar. The species assignment was verified by ITS sequencing. *Ph. infestans* strain 1306, isolated from tomato in San Diego, CA, and later shown to be also pathogenic on potato, was maintained at 18 °C on rye-sucrose agar.

For expression studies on artificial media, rye-sucrose media (recipe A from reference [92]) and pea media [93] were clarified by centrifugation at 5000 ×*g* for 5 min. The resulting broths were then inoculated with 10^4^/ml sporangia of *Ph. infestans* or 2-mm plugs from the edges of *Py. ultimum* cultures. Cultures were harvested at “early” and “late” growth stages, which corresponded to roughly 33% and 100% coverage of the liquid surface, respectively. These were 3-day (nonsporulating) and 5-day (sporulating) cultures of *Ph. infestans* and 1.5 and 3-day cultures of *Py. ultimum.* While hyphal swellings considered to resemble sporangia were detected in *Py. ultimum,* their density was low (<5%) compared to that of *Ph. infestans* sporangia and did not increase with further incubation.

### Plant infections

Tubers (cv. Russet Burbank) were washed in tap water, immersed in 10% (*v*/v) household bleach for 15 min, rinsed in distilled water, cut into 2 mm slices using a mandoline, rinsed in water, and blotted dry. The slices were then placed on a metal rack 8-mm above moist paper towels in a box with a tight-fitting lid.

For inoculations with *Ph. infestans,* zoospores were released from 8-day cultures [94], adjusted to 5 × 10^5^/ml, and about 0.2 ml was spread on the upper surface of each tuber slice using a rubber policeman. Slices were kept at 18 °C in the dark and frozen in liquid nitrogen after 1.5, 2.5, and 4 days. Since *Py. ultimum* does not readily produce zoospores, infections were performed by placing a 2-mm plug from the growing edge of a 2-day V8 agar culture in the center of the slice. After 1.5 days, tuber tissue was excised as described in Results. For each sample, multiple tuber slices were pooled, with biological replicates (triplicates) performed in separate weeks.

### Gene annotation and ortholog analysis

Although annotated gene sets for the two pathogens were available, to allow for valid comparisons these were reanalyzed as described below. The number of genes reported for each functional category may therefore vary slightly from other published studies. Functional annotations were obtained by searching the SwissProt and PANTHER databases, using an *E*-value cut-off of 10^−10^; the results are shown in Additional file 2: Tables S7 and S8. Proteins predicted by both SignalP v4.1 and TargetP v1.1 [95] to contain signal peptides were classified as secreted, excluding those predicted by TMHMM [96] to contain a transmembrane domain after the signal peptide cleavage site or, in the case of CWDEs, predicted by PredGPI [97] to have GPI anchors.

Orthologs were identified by using results from OrthoMCL [66], InParanoid [67] using default settings, and reciprocal best hit using a minimum *E*-value in BlastP of 10^−20^; an assignment of orthology was made only when all three methods gave consistent results. To reduce errors resulting from erroneous gene models, ortholog pairs that varied in size by more than 50% were excluded from the expression analyses presented in Results. Also excluded from the analyses were genes that had ≥98% DNA identity with another gene, to reduce errors in assigning RNA-seq reads to the correct paralog. Phylogenetic analyses were performed using protein alignments generated by MUSCLE [98]. In the case of Additional file 10: Figure S6, these were trimmed using TCS [99] with minimal and maximum filtering options of four and nine, respectively. Trees were then generated using PhyML [100] using the LG substitution model with branch support obtained using the SH-like aLRT option. Branch support was also obtained using MrBayes 3.6 [101] with 500,000 generations, sampling every 200 cycles, 125,000 burn-in cycles, gamma distributed variation, and four heated chains.

### RNA-seq analysis

RNA was obtained by grinding tissue to a powder under liquid nitrogen, followed by extraction using Sigma and Agilent Plant RNA kits for the mycelia and tubers, respectively. A preliminary assessment of disease progression was performed by separating RNA on agarose gel and comparing the ratio of plant to pathogen RNA, taking advantage of the fact that oomycete rRNA is larger than plant rRNA. RNA quality was then assessed using an Agilent 2100 Bioanalyzer.

Indexed libraries for sequencing were then prepared using the Illumina Truseq kit v2. Paired-end libraries were quantitated by Qubit analysis, multiplexed and sequenced on an Illumina HiSeq2500, except for the 1.5-day tuber sample which was sequenced on an Illumina NextSeq500. Reads passing the quality filter were aligned to each pathogen’s genome using Bowtie 2.2.5 and Tophat 2.0.14, allowing for one mismatch. This used the reference genomes of *Ph. infestans* isolate T30–4 and *Py. ultimum* isolate DAOM BR144 [17, 18].

Expression and differential expression calls were made with edgeR using TMM normalization, a generalized linear model, and FDR calculations based on the Benjamini-Hochberg method [102]. Hierarchical clustering, heatmap generation, and PCA analysis were performed using Partek Genomics Suite. GO term enrichment analysis was performed using the GOHyperGAll script [103] with a FDR cut-off of 10^−3^.

### RT-qPCR

Primers (Additional file 2: Table S9) were designed to amplify bands of about 150 bp from the 3′ end of genes. To develop standard curves for determining expression levels, targets were amplified from genomic DNA, resolved by electrophoresis, purified, and quantified using a spectrophotometer. cDNA was prepared using the Maximal First-Strand RT-PCR Kit (Thermo Scientific) using DNAse-treated RNA as template. Control amplifications using no reverse transcriptase confirmed the effectiveness of the DNAase treatment. cDNA (25–50 ng) and serial dilutions of standards (10^1^ to 10^7^ copies) were then subjected to analysis using a CFX Connect System (Biorad) using DyNAmo HS SYBR Green qPCR kit (Thermo Scientific), using triplicate reactions. Cycling parameters consisted of 95 °C for 15 min, followed by 40 three-step amplification cycles consisting of 10 s at 94 °C, 20 s at 55 °C, and a 15 s at 72 °C. Melt curves were generated to verify amplification specificity. For analyses of mRNA levels in tubers, calculations included an adjustment to account for the fraction of mRNA from the pathogens, based on the fraction of reads mapping to their genomes from tubers compared to pure culture in the RNA-seq analysis.

## Additional files

Additional file 1: Table S1. RNA-seq statistics. (PDF 39 kb)

Additional file 2: Table S2. CPM values of all genes. **Table S5.** Pseudogene candidates from *Ph. infestans* and *Py. ultimum.* **Table S6.** Species-specific genes from *Ph. infestans* and *Py. ultimum.* **Table S7.** Revised annotations of *Ph. infestans* genes. **Table S8.** Revised annotations of *Py. ultimum* genes. **Table S9.** Primers used for RT-qPCR. (XLSX 4887 kb)

Additional file 3: Figure S1. Expression of genes involved in secretion. The format of the figure is the same as in Fig. 4. The panels include (top to bottom) orthologs of genes shown necessary for secretion in *Saccharomyces cerevisiae* [46]*,* SNARE genes, and genes encoding RAB GTPases. (TIFF 603 kb)

Additional file 4: Figure S2. Relative sizes of OrthoMCL families. The bars indicate the ratio of the number of genes per family in *Ph. infestans* divided by the number of genes in *Py. ultimum*. (TIFF 208 kb)

Additional file 5: Figure S3. Relationship between expression level and size of gene families. Families were defined by OrthoMCL in the two species. Shown is the summed FPKM of the genes within each family, based on the early rye media samples. (TIFF 325 kb)

Additional file 6: Figure S4. Fold-change scatterplots of orthologs. Plotted are the ratios of CPM values of *Ph. infestans* and *Py. ultimum* orthologs in **a,** early media (numerator) versus early tuber (denominator) and **b,** early tuber (numerator) versus late tuber (denominator). Dashed lines mark three-fold differential expression, i.e. 1.5 in log_2_ values. Genes were included only if their CPM was ≥1 in both conditions, therefore 4739 and 4993 genes are shown in panels **a** and **b**, respectively. The categories represented are: genes up-regulated in both organisms (green spots, zone i), down-regulated in both organisms (orange, zone v), up-regulated in *Py. ultimum* and down-regulated in *Ph. infestans* (red, zone iii), up-regulated in *Ph. infestans* and down-regulated in *Py. ultimum* (brown, zone vii), and showing less than a three-fold change in both species (yellow, zone ix). Cases where orthologs were differentially expressed in only one species are represented by blue, ii; plum, iv; cyan, vi; and magenta, viii. The CPM values were normalized based on the entire gene set, prior to removing non-orthologs. (TIFF 847 kb)

Additional file 7: Table S3. Genes from the zones shown in Additional file 6: Figure S4a. Each of the nine tabs shows the genes and their fold-change levels in each sector in early tubers and media. (XLSX 369 kb)

Additional file 8: Table S4. Genes from the zones shown in Additional file 6: Figure S4b. Each of the nine tabs shows the genes and their fold-change levels in each sector in late and early tuber. (XLSX 381 kb)

Additional file 9: Figure S5. Quantification of representative ortholog pairs with divergent expression using RT-qPCR. mRNA levels are presented as FPKM from RNA-seq, or mRNA copies per pg cDNA from RT-qPCR. Values from RT-qPCR from tubers were corrected for the proportion of RNA estimated to come from the pathogen, based on read mapping statistics from RNA-seq. The orthologous gene pairs were as follows: **a,** anthranilate synthase (PITG_01711 and PYU1_G005607) in late tubers. **b,** anthranilate phosphoribosyltransferase (PITG_17032, PYU1_G006488) in late tubers. **c,** 20G–Fe(II) oxygenase (PITG_00237, PYU1_G003898) from early tubers. **d,** 12-oxophytodienoate reductase (PITG_08491, PYU1_G007433) from early pea. **e,** 20G–Fe(II)oxygenase (PITG_08301, PYU1_G002215) from early pea. **f,** carboxypeptidase (PITG_00756, PYU1_G014383) from early tubers. (TIFF 360 kb)

Additional file 10: Figure S6. Features of MACPF/perforin from *Py. ultimum.* **a,** structure of predicted protein. **b,** expression pattern in media and tubers. **c,** phylogenetic analysis of selected MACPF-containing proteins from *Pythium* and other taxa. Shown is a PhyML tree, with values at nodes representing SH-like aLRT values above 70 from PhyML, and posterior probability values above 90 from mrBayes. Accession numbers of sequences are *A. thaliana* AAG51760.1, *B. bigemina* CDR97760.1, *C. owczarzaki* XP_004348917.1, *C. gigas* XP_011434062.1, *D. misasensis* WP_034336981.1, *E. tenella* XP_013228726.1, *G. gallus* AGL75461.1, *G. japonicus* XP_015262306.1, *H. sapiens* CAA31612.1, *M. virescens* SDD87368.1, *P. vivax* KMZ87443.1, *P. trichocarpa* XP_002305709.1, *Py. arrhenomanes* PAR_G002296, *Py. irregulare* PIR_G000461, *Py. ultimum* PYU1_G000106, *S. lycopersicum* XP_004232984.1, *T. thermophila* XP_001019028.1, *T. parva* XP_765691.1, and *T. gondii* KFH05725.1. Accession numbers are from GenBank except for *Pythium* spp., which are gene names from fungidb.org. (TIFF 329 kb)

## Abbreviations

AAAP: Amino acid/auxin permease
AMT: Ammonium transporter
APC: Amino acid/polyamine/organocation transporter
CPM: Counts per million mapped reads
CRN: Crinkler effector
CWDE: Cell wall-degrading enzyme
DAAC: Dicarboxylate/amino acid:cation symporter
DPI: Days post-infection
FDR: False discovery rate
FPKM: Fragments per kilobase per million mapped reads
GO: Gene ontology
GPH: Glycoside-pentoside-hexuronide transporter
GPI: Glycosylphosphatidylinositol
GST: Glutathione S-transferase
MACPF: Membrane-attack complex/perforin
PCA: Principal component analysis
TMM: Trimmed mean of M-values
