# Supplementary material for: Lifestyle, gene gain and loss, and transcriptional remodeling cause divergence in the transcriptomes of Phytophthora infestans and Pythium ultimum during potato tuber colonization
Source: BMC Genomics. 2017 Oct 10;18:764. doi: 10.1186/s12864-017-4151-2 (PMC5635513; doi:10.1186/s12864-017-4151-2)
Supplement: Supplementary file 1 — RNA-seq statistics. (PDF 39 kb) [file 12864_2017_4151_MOESM1_ESM.pdf]

Table S1. RNA-seq statistics.

Species	Condition	Biological replicates	Total reads	Reads mapped to pathogen	Per cent mapped
Ph. infestans	Rye early	3	321,535,026	287,788,693	89.5
	Rye late	3	271,184,730	240,519,772	88.7
	Pea early	3	299,350,646	270,669569	90.4
	Pea late	3	303,352,320	270,782,855	89.3
	Tuber early	3	1,050,012,734	40,645,641	3.9
	Tuber middle	3	1,266,994,058	383,656,380	30.3
	Tuber late	3	927,870,736	708,776,144	76.4
Py. ultimum	Rye early	3	230,807,200	216,528,025	93.8
	Rye late	3	216,908,728	203,271,994	93.7
	Pea early	3	208,553,786	194,253,631	93.1
	Pea late	3	186,757,304	173,097,089	92.7
	Tuber early	3	461,099,278	113,754,907	24.7
	Tuber middle	3	207,358,216	182,141,252	87.8
	Tuber late	3	246,459,330	222,079,444	90.1
