# Supplementary figures and images for: Lifestyle, gene gain and loss, and transcriptional remodeling cause divergence in the transcriptomes of Phytophthora infestans and Pythium ultimum during potato tuber colonization
Source: BMC Genomics. 2017 Oct 10;18:764. doi: 10.1186/s12864-017-4151-2 (PMC5635513; doi:10.1186/s12864-017-4151-2)

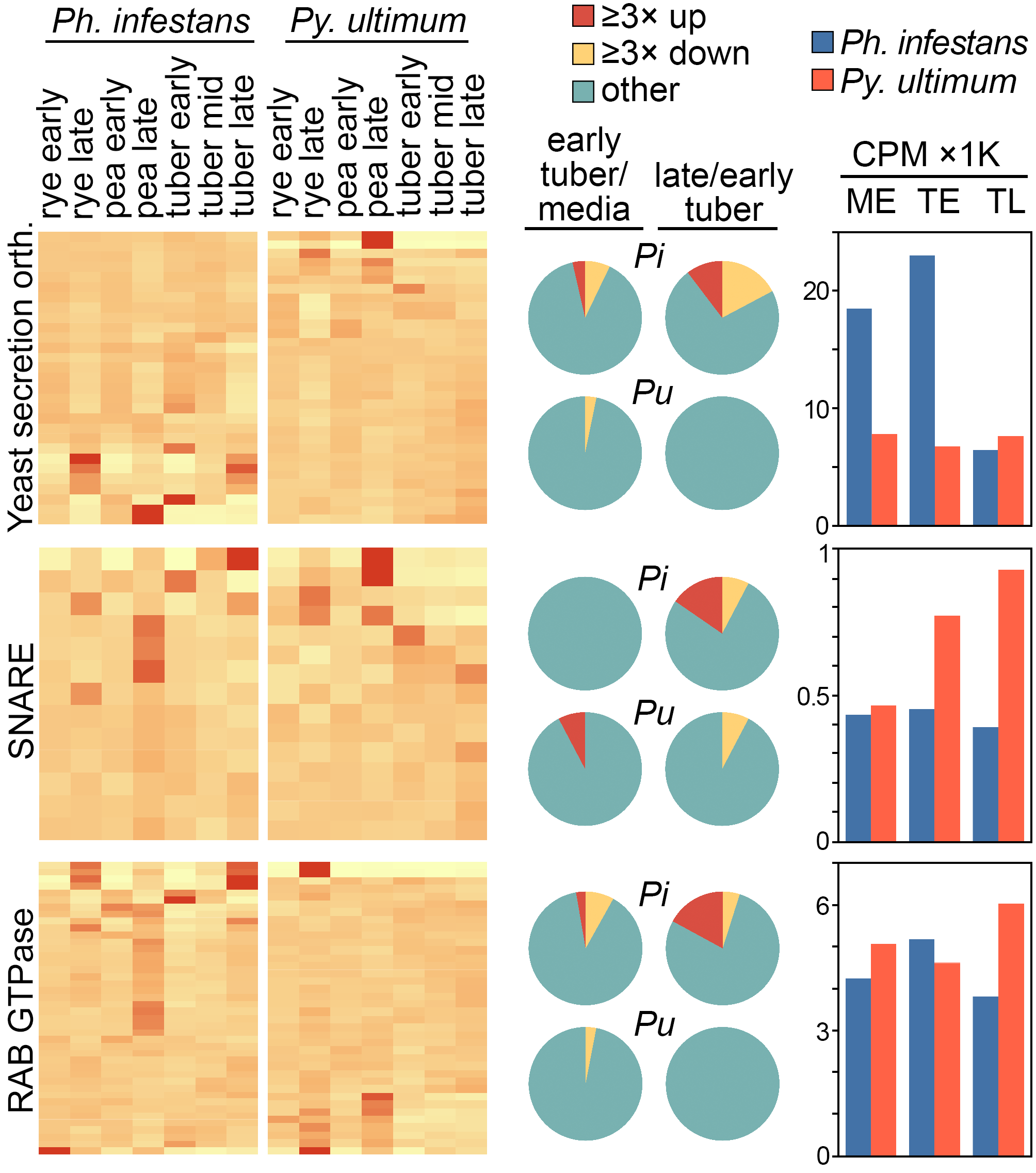

Supplement: Supplementary file 3 — Expression of genes involved in secretion. The format of the figure is the same as in Fig. 4. The panels include (top to bottom) orthologs of genes shown necessary for secretion in Saccharomyces cerevisiae [46], SNARE genes, and genes encoding RAB GTPases. (TIFF 603 kb) [file 12864_2017_4151_MOESM3_ESM.tif]

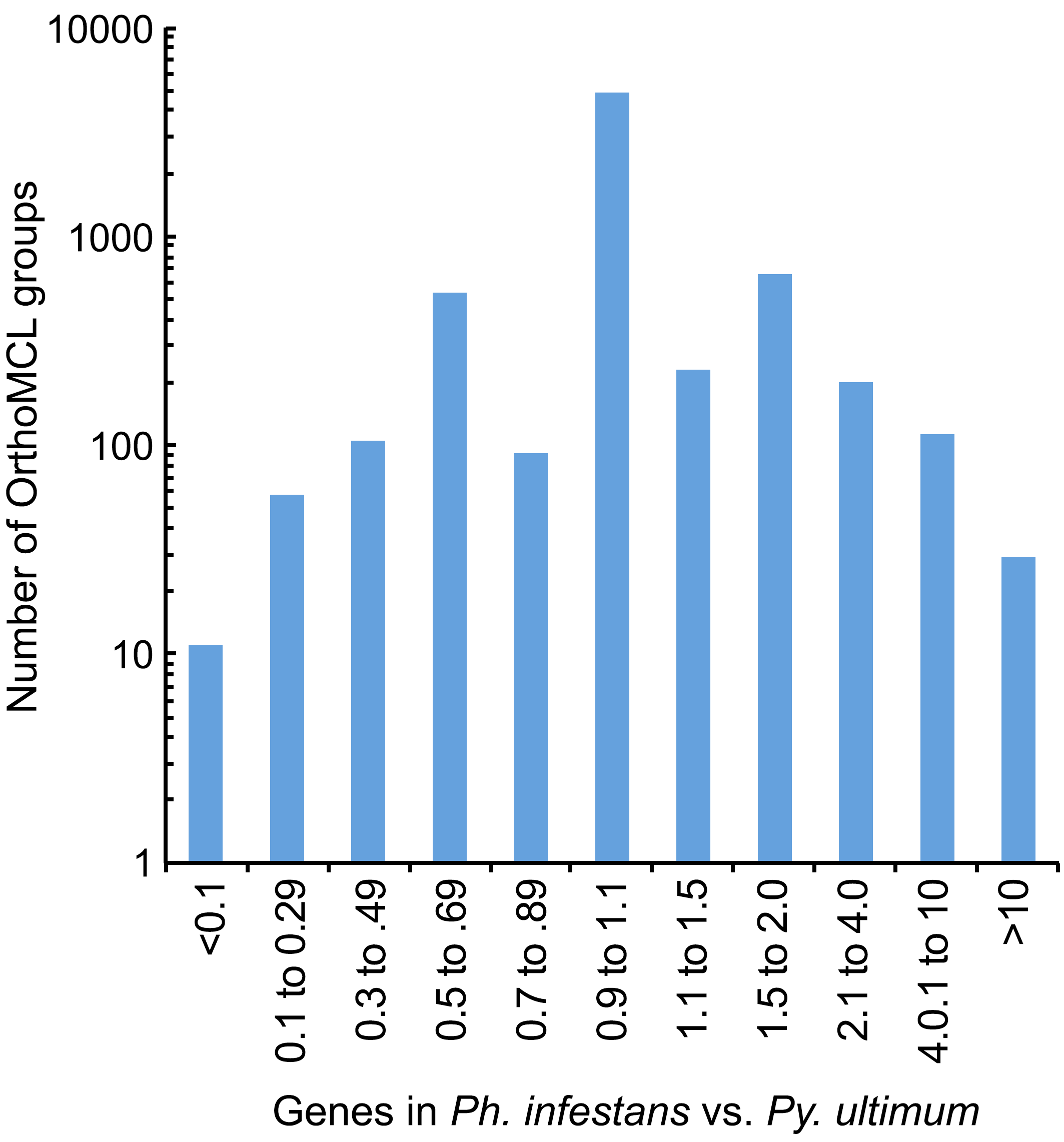

Supplement: Supplementary file 4 — Relative sizes of OrthoMCL families. The bars indicate the ratio of the number of genes per family in Ph. infestans divided by the number of genes in Py. ultimum. (TIFF 208 kb) [file 12864_2017_4151_MOESM4_ESM.tif]

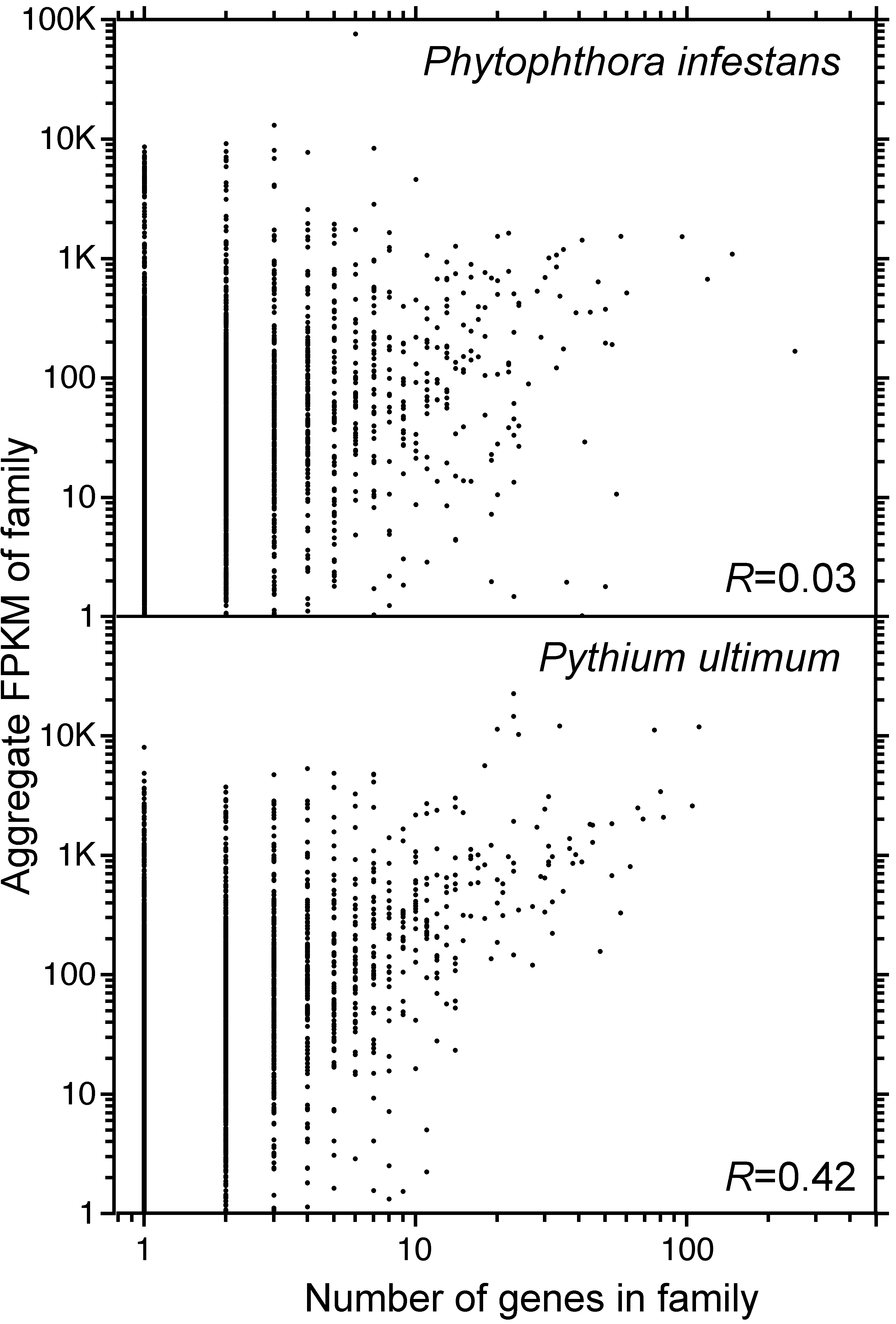

Supplement: Supplementary file 5 — Relationship between expression level and size of gene families. Families were defined by OrthoMCL in the two species. Shown is the summed FPKM of the genes within each family, based on the early rye media samples. (TIFF 325 kb) [file 12864_2017_4151_MOESM5_ESM.tif]

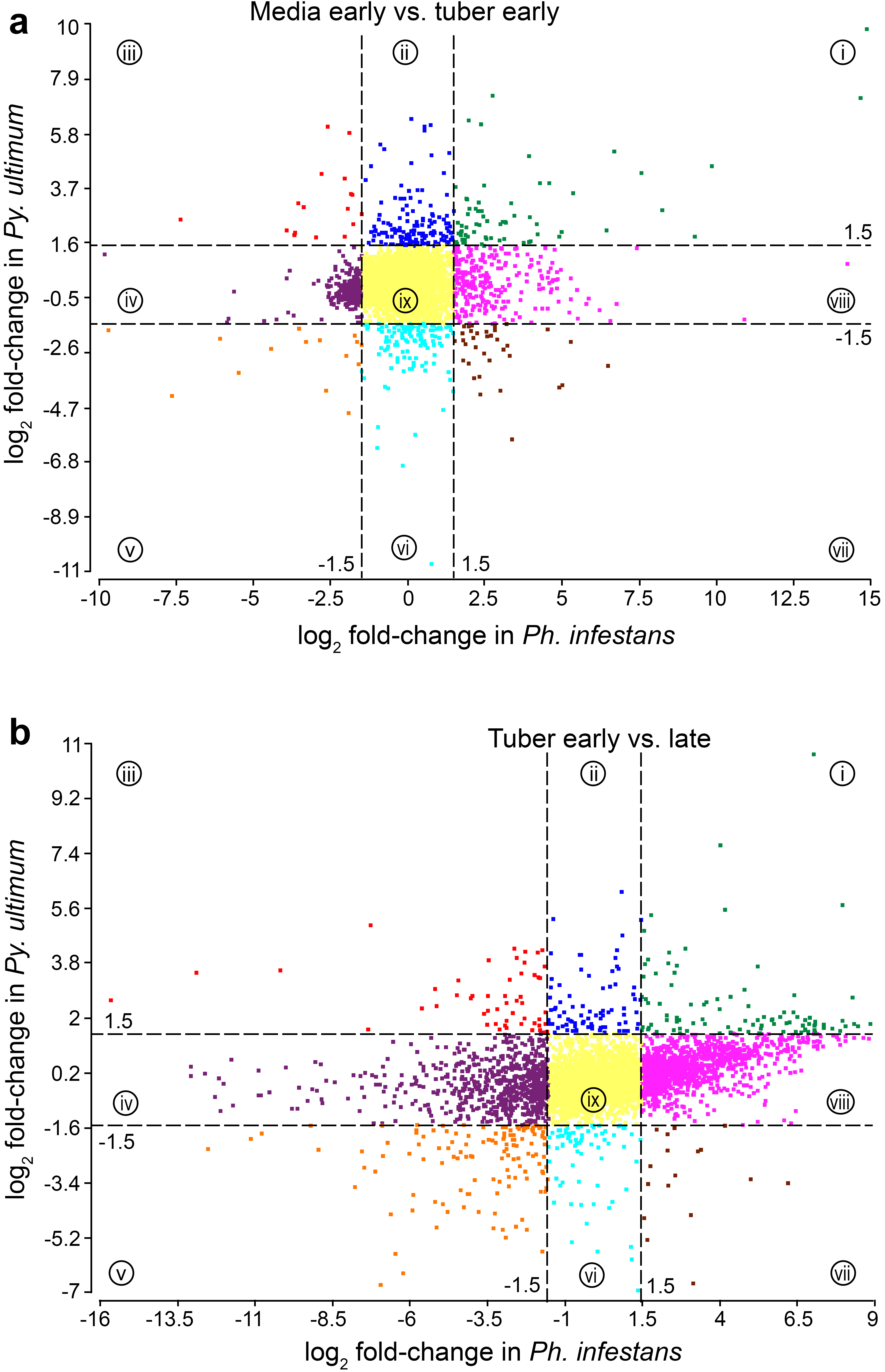

Supplement: Supplementary file 6 — Fold-change scatterplots of orthologs. Plotted are the ratios of CPM values of Ph. infestans and Py. ultimum orthologs in a, early media (numerator) versus early tuber (denominator) and b, early tuber (numerator) versus late tuber (denominator). Dashed lines mark three-fold differential expression, i.e. 1.5 in log2 values. Genes were included only if their CPM was ≥1 in both conditions, therefore 4739 and 4993 genes are shown in panels a and b, respectively. The categories represented are: genes up-regulated in both organisms (green spots, zone i), down-regulated in both organisms (orange, zone v), up-regulated in Py. ultimum and down-regulated in Ph. infestans (red, zone iii), up-regulated in Ph. infestans and down-regulated in Py. ultimum (brown, zone vii), and showing less than a three-fold change in both species (yellow, zone ix). Cases where orthologs were differentially expressed in only one species are represented by blue, ii; plum, iv; cyan, vi; and magenta, viii. The CPM values were normalized based on the entire gene set, prior to removing non-orthologs. (TIFF 847 kb) [file 12864_2017_4151_MOESM6_ESM.tif]

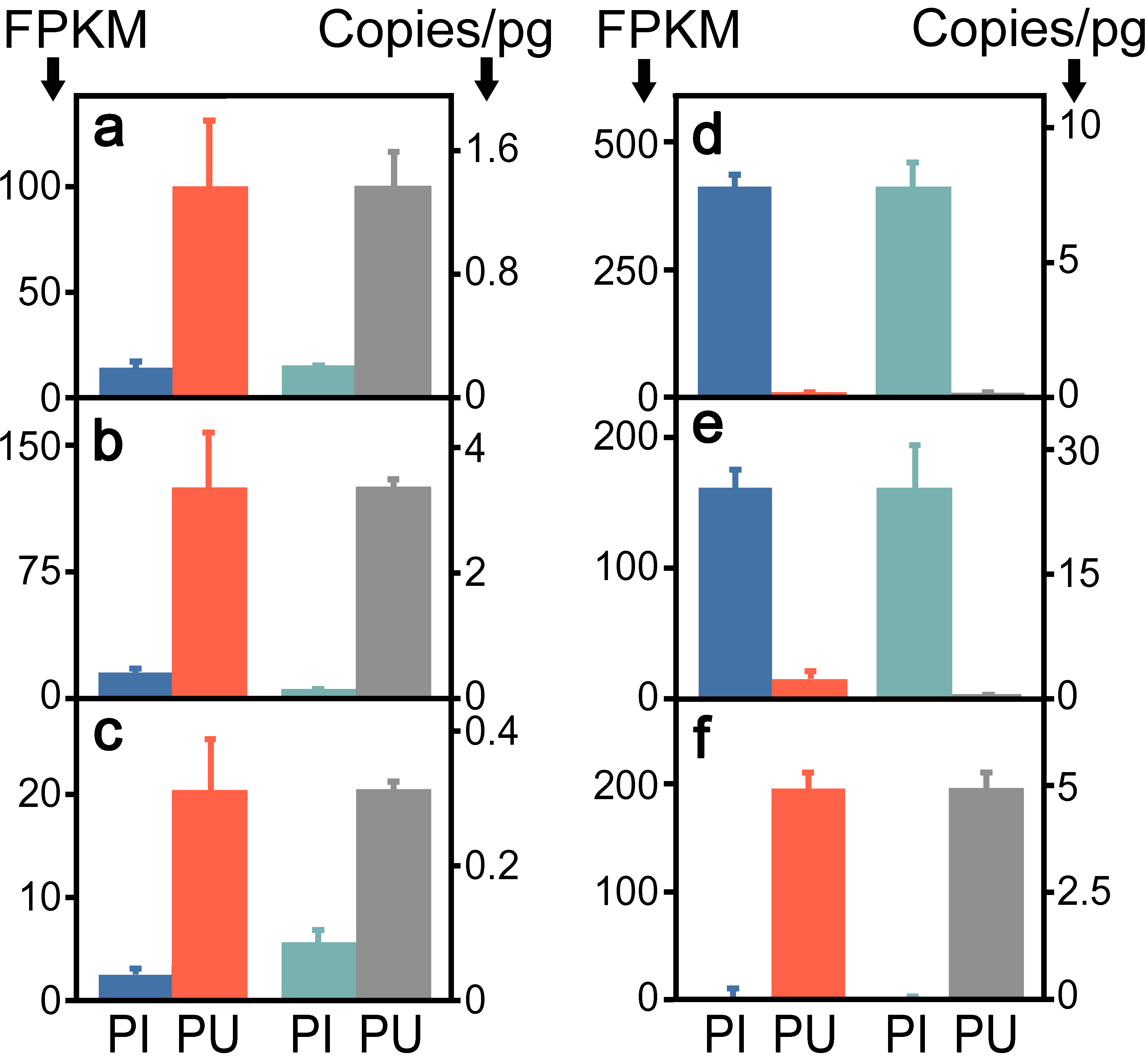

Supplement: Supplementary file 9 — Quantification of representative ortholog pairs with divergent expression using RT-qPCR. mRNA levels are presented as FPKM from RNA-seq, or mRNA copies per pg cDNA from RT-qPCR. Values from RT-qPCR from tubers were corrected for the proportion of RNA estimated to come from the pathogen, based on read mapping statistics from RNA-seq. The orthologous gene pairs were as follows: a, anthranilate synthase (PITG_01711 and PYU1_G005607) in late tubers. b, anthranilate phosphoribosyltransferase (PITG_17032, PYU1_G006488) in late tubers. c, 20G–Fe(II) oxygenase (PITG_00237, PYU1_G003898) from early tubers. d, 12-oxophytodienoate reductase (PITG_08491, PYU1_G007433) from early pea. e, 20G–Fe(II)oxygenase (PITG_08301, PYU1_G002215) from early pea. f, carboxypeptidase (PITG_00756, PYU1_G014383) from early tubers. (TIFF 360 kb) [file 12864_2017_4151_MOESM9_ESM.tif]

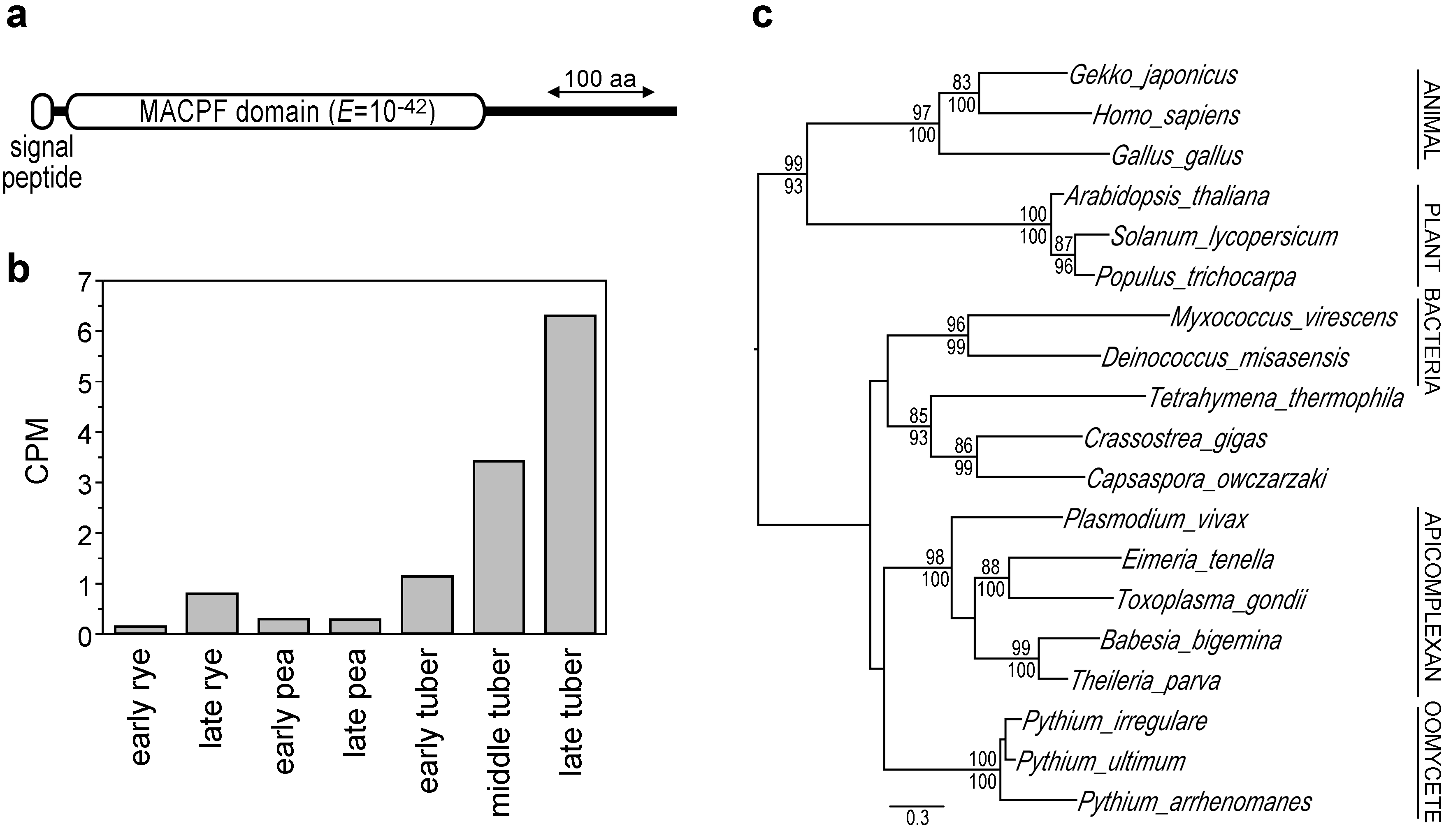

Supplement: Supplementary file 10 — Features of MACPF/perforin from Py. ultimum. a, structure of predicted protein. b, expression pattern in media and tubers. c, phylogenetic analysis of selected MACPF-containing proteins from Pythium and other taxa. Shown is a PhyML tree, with values at nodes representing SH-like aLRT values above 70 from PhyML, and posterior probability values above 90 from mrBayes. Accession numbers of sequences are A. thaliana AAG51760.1, B. bigemina CDR97760.1, C. owczarzaki XP_004348917.1, C. gigas XP_011434062.1, D. misasensis WP_034336981.1, E. tenella XP_013228726.1, G. gallus AGL75461.1, G. japonicus XP_015262306.1, H. sapiens CAA31612.1, M. virescens SDD87368.1, P. vivax KMZ87443.1, P. trichocarpa XP_002305709.1, Py. arrhenomanes PAR_G002296, Py. irregulare PIR_G000461, Py. ultimum PYU1_G000106, S. lycopersicum XP_004232984.1, T. thermophila XP_001019028.1, T. parva XP_765691.1, and T. gondii KFH05725.1. Accession numbers are from GenBank except for Pythium spp., which are gene names from fungidb.org. (TIFF 329 kb) [file 12864_2017_4151_MOESM10_ESM.tif]
